# Genetic Stability and Fitness of *Aedes aegypti* Red-Eye Genetic Sexing Strains With Pakistani Genomic Background for Sterile Insect Technique Applications

**DOI:** 10.3389/fbioe.2022.871703

**Published:** 2022-03-31

**Authors:** Muhammad Misbah-ul-Haq, Danilo O. Carvalho, Lucia Duran De La Fuente, Antonios A. Augustinos, Kostas Bourtzis

**Affiliations:** ^1^ Insect Pest Control Laboratory, Joint FAO/IAEA Centre of Nuclear Techniques in Food and Agriculture, Seibersdorf, Austria; ^2^ Nuclear Institute for Food and Agriculture, Peshawar, Pakistan

**Keywords:** area-wide integrated pest management, vector control, dengue, Zika, insect pest control

## Abstract

The mosquito species *Aedes aegypti* is the primary transmitter of viruses that cause endemic diseases like dengue in Pakistan. It is also a cause of other vector-borne diseases like yellow fever, Zika fever, and chikungunya, which significantly impact human health worldwide. In the absence of efficient vaccines (except for yellow fever) or drugs, vector control methods, such as the sterile insect technique (SIT), have been proposed as additional tools for the management of these diseases. Mosquito SIT programs are based on the release of sterile males and it is important female releases to be ideally zero or to be kept at a minimum, since females are the ones that bite, blood-feed and transmit pathogens. Recently, an *Ae. aegypti* genetic sexing strain (GSS), with and without a recombination-suppressing inversion (Inv35), was developed using the eye color as a selectable marker, with males having black eyes and females red eyes. In the present study, we introgressed the sexing features and the Inv35 of the *Ae. aegypti* red-eye GSS into the Pakistani genomic background aiming to their future use for SIT applications in the country. Both introgressed strains, the Red-eye GSS-PAK and the Red-eye GSS/Inv35-PAK, were evaluated in respect to their genetic stability and biological quality by assessing parameters like recombination rate, fecundity, fertility, pupal and adult recovery, time of development, pupal weight, survival, and flight ability in comparison with a wild Pakistani population (PAK). The results suggest that the sexing features and the recombination suppression properties of Inv35 were not affected after their introgression into the local genomic background; however, some biological traits of the two newly constructed strains were affected, positively or negatively, suggesting that a thorough quality control analysis should be performed after the introgression of a GSS into a new genomic background prior to its use in SIT field trials or applications. The importance of using GSS with local genomic background for SIT applications against *Aedes aegypti* is also discussed.

## Introduction

*Aedes aegypti* mosquitoes are responsible for the transmission of numerous viral infections among humans (Bhatt et al., 2013) in particular considered as a major vector of viruses that are responsible for diseases like dengue, chikungunya, Zika fever, and yellow fever (Morrison et al., 2008; Souza-Neto et al., 2019). It has spread over the world’s tropical and subtropical regions and breeds in artificial containers within human environments to have easy access to blood for feeding and almost no predators (Brown et al., 2011).

Dengue, along with the other infections mentioned above, is becoming a global public health concern due to its rapid geographical spread in parallel (Guzman and Harris, 2015; Kraemer et al., 2015). In Pakistan, dengue has also become more common over recent decades and has been spreading at an alarmingly high rate, with cases being reported from urban and rural areas from different regions of the country (Khan et al., 2018). Numerous factors like climatic changes, public unawareness, inadequate surveillance, and insufficient funding have contributed to frequent dengue outbreaks (Ahmad et al., 2017).

In addition to the reduction of dengue transmission, vector control is also desirable to avoid nuisance and hypersensitivity/allergies mediated by bites (Paris et al., 2011; Bowman et al., 2016; Barrera et al., 2017). Presently, vector control mainly depends on insecticides applied on mosquito larval habitats and against adult mosquitoes indoors and during dengue outbreaks. However, the selective pressure on populations resulting in insecticide resistance has become an issue for chemical control in several *Ae. aegypti* mosquito populations worldwide. Furthermore, only a few new insecticides have been commercialized for dengue vector control (Vontas et al., 2012; Smith, 2016). In many Pakistani field populations of dengue vectors, it is common to find insecticide resistance at moderate to high levels, which has been already reported as a leading future problem regarding vector control (Khan et al., 2011; Arslan et al., 2016). Particularly in urban areas, *Ae. aegypti* has been reported to develop resistance against commonly used insecticides (Jahan and Shahid, 2013).

As conventional control methods are not effective enough, environmentally friendly and species-specific approaches such as the sterile insect technique (SIT) are needed to control mosquito vector populations (Bouyer and Lefrançois, 2014; Carvalho et al., 2014; Lees et al., 2015; Bourtzis et al., 2016). SIT is an insect pest control method which is based on the release of sterile males to suppress, prevent the (re)introduction, contain or even locally eradicate insect pest populations. SIT has been in use for decades as an effective tool to suppress or even eliminate numerous insect pests such as the New World screwworm, tsetse fly, Mediterranean fruit fly etc. (Baumhover, 1966; Ciss et al., 2019; Gutierrez et al., 2019; Dyck et al., 2021b).

SIT is a species-specific and environmentally friendly method to control populations of insect pests and disease vectors (Dyck et al., 2021a). In SIT, radiation sterilizes male mosquitoes, which are released in the open environment to mate with wild females, thus resulting in reduced fertile crosses and subsequent population suppression (Dyck et al., 2021a). A successful SIT mosquito release program’s primary obstacle is eliminating or separating the females because, in this case, only females bite and transmit the etiological agent. Therefore, removing females prior to sterile males’ release is a strict prerequisite (Gilles et al., 2014; Papathanos et al., 2018; Lutrat et al., 2019).

Sex separation strategies currently available are time- and labor-consuming, and highly prone to errors. Efficient and robust sex separation methods are not yet fully available in mass-rearing facilities (Gilles et al., 2014; Papathanos et al., 2018; Zacarés et al., 2018; Lutrat et al., 2019; Zheng et al., 2019; Crawford et al., 2020). In addition, genetic and molecular-based approaches can be exploited for the development of more convenient, reliable, efficient and cost-effective methods for mosquito sex separation at a mass-rearing scale (Gilles et al., 2014; Papathanos et al., 2018; Lutrat et al., 2019). For example, genetic sexing strains (GSS) with phenotypic markers to distinguish male from female mosquitoes may prove useful. An excellent example of GSS developed and reared in mass rearing facilities worldwide for SIT purposes are the VIENNA 7 and VIENNA 8 GSS of the Mediterranean fruit fly *Ceratitis capitata*, which are based on a color and a thermal lethality mutation linked to the sex (Augustinos et al., 2017; Franz et al., 2021).

Recently, such a GSS for *Ae. aegypti* was developed through classical genetics by exploiting the red-eye mutation (*re*) as a phenotypical marker, resulting in females with red eyes and males with black eyes through all the developmental stages (Koskinioti et al., 2021). However, this strain still had recombinants, which would compromise the genetic stability and the GSS efficiency. A radiation-induced chromosomal inversion (Inv35) was then introduced as a recombination suppressor to enhance its genetic stability (Augustinos et al., 2020). Through laboratory-scale quality control tests, it was evident that the strain exhibited sufficient biological quality to be considered as a candidate for *Ae. aegypti* SIT programs (Koskinioti et al., 2021). In a subsequent study it was shown that the recombination frequency in the GSS strains, with and without the inversion, is not affected if the red-eye mutation and the Inv35 are introduced to six different genomic backgrounds, Brazil, Indonesia, Mexico, Sri Lanka, Singapore, and Thailand (Augustinos et al., 2022).

However, it is known that the background genotype contributes significantly to the biological quality and the performance of insect strains aimed for releases especially in terms of mating success (Quintero-Fong et al., 2016; Carvalho et al., 2020; Leftwich et al., 2021). Laboratory reared insects differ from wild ones due to combined effects of bottlenecking, high inbreeding, selection for artificial rearing, and genetic variation. In the present study, we introgressed the red-eye mutation and the chromosomal inversion Inv35 into the local (Pakistani) genomic background and the two new GSS developed, Red-eye GSS-PAK and the Red-eye GSS/Inv35-PAK were evaluated in respect to their genetic stability, biological quality, and their potential to be used for SIT applications against *Ae. aegypti* populations in Pakistan.

## Materials and Methods

### *Aedes aegypti* Strains and Rearing *Conditions*

The Rexville red eye mutant strain used in the present study is a long-domesticated laboratory strain (kindly provided by Dr. Margareth Capurro at the Department of Parasitology, University of Sao Paulo, Brazil) and had previously been used in other studies including the construction of the *Ae. aegypti* red-eye GSS (Costa-da-Silva et al., 2017; Augustinos et al., 2020, 2022). The color of both compound and simple eye of this strain remains red throughout development. The chromosomal inversion (Inv35) was induced through irradiation, and it is known to suppress recombination between the red eye and M loci (Augustinos et al., 2020; Koskinioti et al., 2021). PAK is a recently domesticated strain from mosquitoes collected from Northern areas of Pakistan’s KP Province and was used as a source of the local genomic background. The introgression of the red eye mutation and Inv35 was performed as described previously (Augustinos et al., 2022), consisted of a series of backcrosses and was expected to result into two new GSS, Red-eye GSS-PAK and the Red-eye GSS/Inv35-PAK with ∼98.8 and ∼98% PAK genetic background, respectively. The two new GSS were evaluated for their genetic stability until the seventh generation while their biological quality was assessed at the third generation in the present study.

All strains were kept under standard rearing conditions (FAO-IAEA, 2017). More specifically, mosquitoes were maintained in the insectary of the Insect Pest Control Laboratory of the Joint FAO/IAEA Centre of Nuclear Techniques in Food and Agriculture, Seibersdorf, Austria, at 27 ± 1°C, 80% RH, and a photoperiod of 12/12 h day/night, including 1 h of twilight. Females of all strains were blood-fed for 20 min, two times per day for two consecutive days per week, using collagen casing with porcine blood. In addition, 10% sugar solution was provided continuously in 30 × 30 × 30 cm adult plastic cages (BugDorm-1, MegaView Science Co., Taiwan). Eggs were collected by keeping moistened white filter paper in urine cups half-filled with water at least 72 h after blood feeding.

### Genetic Stability

In each generation, after sorting pupae in glass pupal sorter, a minimum of 1,000 male and female pupae were screened under a common stereomicroscope. Both expected and recombinant genotypes were counted separately in male and female pupae by observing the genital lobe and the eye color. Data was recorded in the appropriate spreadsheet for each generation. For strains maintained under filtering, males with red eyes and females with black eyes (recombinants) were counted and removed from the colonies (Koskinioti et al., 2021). For non-filtered colonies, and following counting recombinants, all insects were transferred in the same cage to set up the next generation.

### Biological Quality

Red-eye GSS-PAK and Red-eye GSS/Inv35-PAK, both at the third generation, were compared with a recently domesticated PAK strain in respect to the following biological quality parameters:

#### Fecundity

For each strain, 50 newly emerged males and an equal number of females were released together in a plastic rearing cage (BugDorm-1 rearing cage 30 × 30 × 30 cm) and mated for 3–4 days. Pre-mated females were blood-fed twice per day for 20 min for two consecutive days to ensure full engorgement. Three replicates of 10 fully fed females per small cage (BugDorm-4S1515 with 15 × 15 × 15 cm) were performed for each strain. Eggs were collected for the first two gonotrophic cycles. Dead females (if any) were replaced by other gravid females of the same age. Eggs were counted under a common stereomicroscope before drying. The total number of eggs was divided by 10 to estimate the average number of eggs per female per replicate.

#### Fertility

Eggs from the fecundity test of each replicate from both gonotrophic cycles were hatched by placing egg papers in airtight glass jars (500 ml), prepared in advance to have water with low dissolved oxygen content (boiled water), and 2–4 drops of larval diet were added to stimulate egg hatching. Jars prepared in the morning were kept in an incubator for hatching at 27°C until the next day (around 24 h). First instar larvae (L1) were counted by aspirating with a 200 μl tip on the plastic pipette. The percentage of hatching was recorded of all three strains.

#### Recovery Rates and Development Time

Pupal recovery and adult recovery were recorded by counting the total number of pupae and adults respectively, deriving from the total number of eggs. Development time was recorded by counting the number of pupae of each sex collected for each strain every 24 h. The duration of development was estimated from egg hatching to pupation.

#### Pupal Weight

Ten female and 10 male pupae per replicate were slightly air-dried for 20 min by placing them on a towel paper, observing and shaking the trays until they are not clustered. Batches of 10 pupae each were weighed to calculate the average pupal weight. In total, five replicates were counted for each strain per sex.

#### Survival Rate

Fifty newly emerged males and females per replicate per strain were kept in small cages BugDorm-4S1515 (15 × 15 × 15 cm). Each cage was provided with 10% sucrose solution. Dead mosquitoes were counted and removed daily. At the end of a 33-day period, dead mosquitoes were counted and subtracted from the total number of adults released to estimate the average survival rate. Three replicates were made per sex per strain.

#### Flight Ability

Approximately one hundred 4–5 days old adult males of each strain per replicate were tested in a Flight Test Device (FTD) as described previously (Culbert et al., 2018). Three replicates per strain were performed. After 2 h from the release time, successful fliers were aspirated from the outer part of the FTD and were counted. Similarly, unsuccessful fliers trapped in the glass tubes and the releasing arena were also counted. The number of successful fliers out of the total number of adult males released corresponded to the flight ability percentage.

### Statistical Analysis

All statistical analysis was performed using R language 4.1.2—“Bird Hippie” (R Core Team, 2020) with RStudio environment—version 2021.09.02 + 382 (RStudio Team, 2016). Normality was assessed by the data frequency distribution and its point distribution of the quantile-quantile plot, and it was then determined whether parametric or not. The alpha < 0.05 was considered statistically significant for all generalized linear models used for each parameter evaluated with a multiple comparison of the mean using Tukey contrasts as post hoc. The model has considered binomial distribution for percentages Poisson distributions for counting, considering logit and log their respective transformations. The box and whiskers plot were used to demonstrate the full data distribution representing the minimum, maximum, median, 1st and 3rd quartiles. Parametric statistical comparisons were only performed using multiple comparisons of means by computing the contrast matrices of all comparisons obtained by each generalized linear model. For survival analysis, the Kaplan-Meier, Log-rank test, and the Cox proportional-hazards model were used to distinguish differences and also to obtain the survival curve plot using the *survival* package (Therneau, 2020). Information about additional packages used in the present study can be found in the supporting material together with all statistical analysis (Supplementary Material S1) and the original data used for all analysis (Supplementary Material S2).

## Results

### Genetic Stability

**FIGURE 1:**
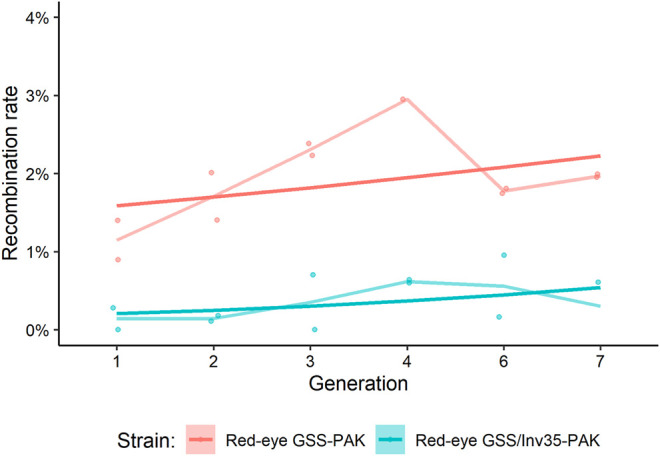
Recombination rate in Red-eye GSS-PAK and Red-eye GSS/Inv35-PAK strains during seven generations. Lighter lines represent the mean of the points, and the darker straight lines represent the GLM line for each strain.

The genetic stability was assessed by recording the expected and recombinant genotypes in the Red-eye GSS-PAK and the Red-eye GSS/Inv35-PAK strains up to the seventh generation. In total, 10,211 Red-eye GSS-PAK and 8,479 Red-eye GSS/Inv35-PAK individuals were screened and the recombination rate ranged between 1.15–3.70% and 0.14–0.62%, respectively (Supplementary Material S2). The results presented in Figure 1 confirm that the presence of Inv35 significantly suppresses the recombination rate (Df = 1, F = 25.73, *p* = 2.6^−6^) (Figure 1).

### Biological Quality

#### Fecundity

**FIGURE 2:**
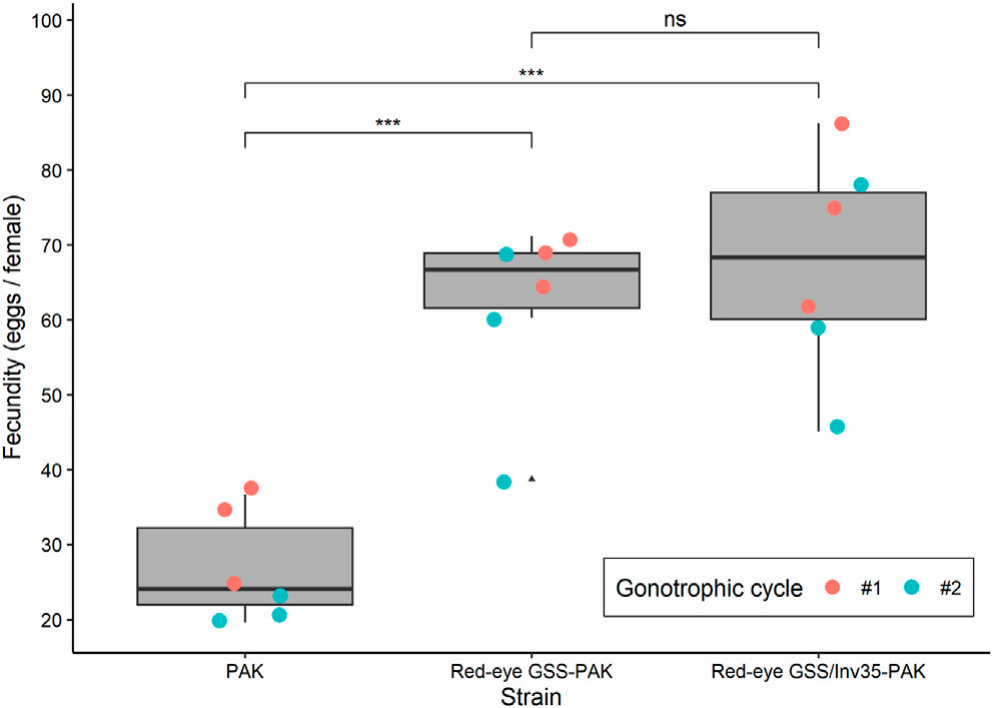
Fecundity of the PAK, Red-eye GSS-PAK, and Red-eye GSS/Inv35-PAK strains during the first and second gonotrophic cycle. Significance symbols: “***” for *p* < 0.001, and “ns” for “not significant”.

The introduction of the red eye mutation and the inversion Inv35 into the Pakistani genomic background had a positive impact on the fecundity, which was significantly increased in the first and the second gonotrophic cycle in both GSS. The average fecundity of the PAK strain was 31.97 and 21.60 eggs/female in the first and the second gonotrophic cycle, respectively. On the contrary, the average fecundity of the Red-eye GSS-PAK was 68.60 and 55.67 eggs/female while that of the Red-eye GSS/Inv35-PAK was 74.27 and 60.80 eggs per female in the first and the second gonotrophic cycle, respectively, significantly higher than the values recorded for the PAK strain (Df = 2, F = 26.862, *p* = 0.00257—Figure 2). There was no statistically significant difference in the fecundity between the Red-eye GSS-PAK and Red-eye GSS/Inv35-PAK strains (z = −1.94 *p* = 0.127), while the fecundity of both was higher than that of the PAK strain (z = 8.724 and 7.216 with *p* = < 1^−4^, for Red-eye GSS-PAK and Red-eye GSS/Inv35-PAK respectively).

#### Fertility

**FIGURE 3:**
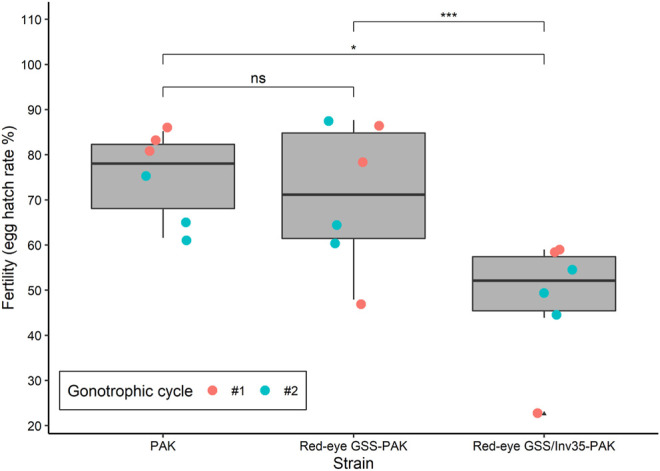
Fertility of the PAK, Red-eye GSS-PAK, and Red-eye GSS/Inv35-PAK strains. Significance symbols: “***” for *p* < 0.001, “*” for *p* < 0.05, “ns” for “not significant”.

Significant differences were observed among the three strains (PAK, Red-eye GSS-PAK and Red-eye GSS/Inv35-PAK) in respect to the fertility (egg hatching) (Df = 2, F = 6.062, *p* = 0.00799—Figure 3). The average egg hatching of the PAK strain was 75.24%, slightly reduced in Red-eye GSS-PAK to 70.92% with no statistic difference (z = −0.37, *p* = 0.92584), while more pronounced reduction was observed in the Red-eye GSS/Inv35-PAK 48.04% (PAK: z = −2.38 *p* = 0.04367, and Red-eye GSS-PAK: z = −3.12, *p* = 0.00487).

#### Pupal and Adult Recovery Rates

**FIGURE 4:**
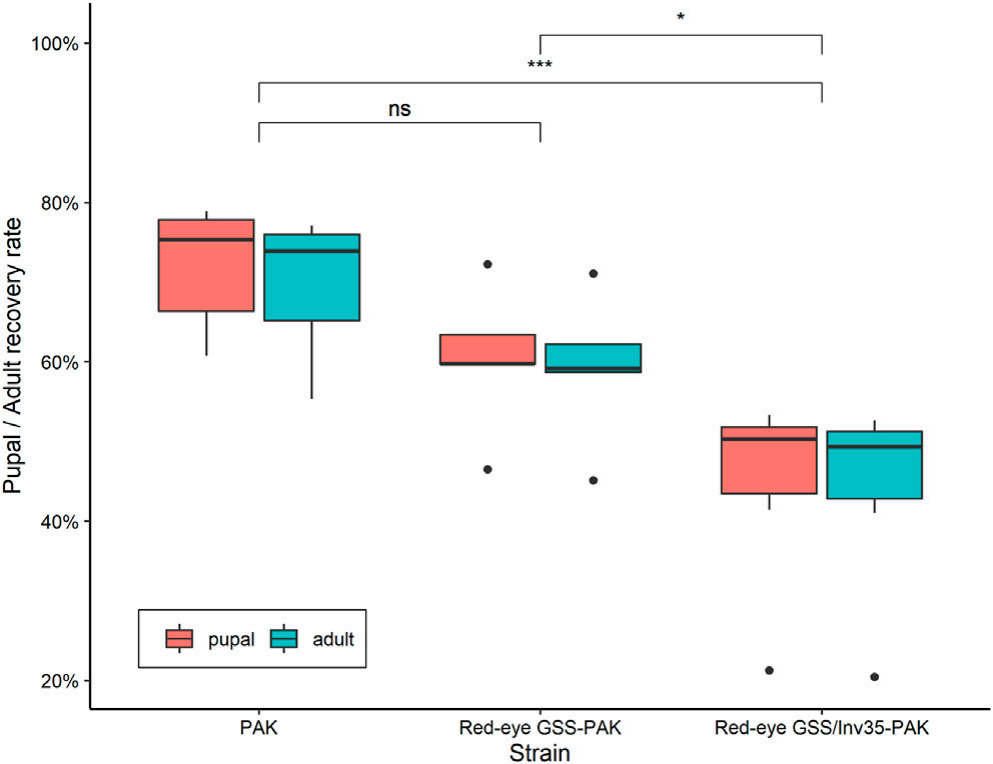
Pupal and adult recovery rate of the PAK, Red-eye GSS-PAK, and Red-eye GSS/Inv35-PAK strains. Significance symbols: “***” for *p* < 0.001, “*” for *p* < 0.05, and “ns” for “not significant”.

Statistically significant difference was observed among the strains in respect to the pupal and adult recovery rates. The average pupal and adult recovery were 72 and 70% in the wild type PAK strain, 60 and 59% in the Red-eye GSS-PAK strain, and 45 and 44% in the Red-eye GSS/Inv35-PAK strain, respectively (Pupal: Df = 2, F = 11.18, *p* = 0.00125; Adult: Df = 2, F = 9.434, *p* = 0.00254—Figure 4).

**FIGURE 5:**
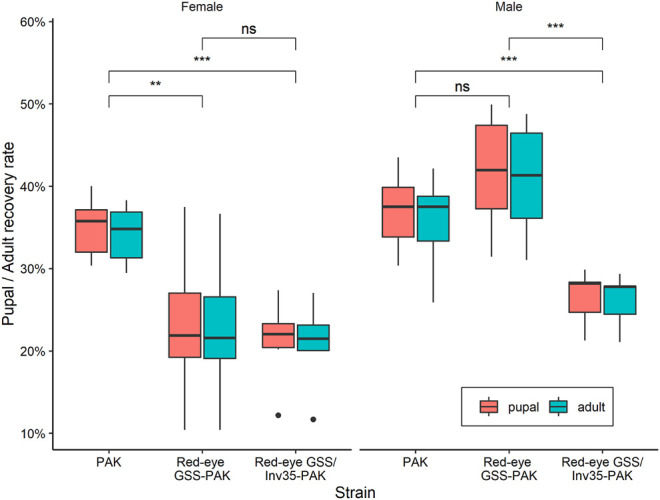
Pupal and adult recovery rate of males and females of the PAK, Red-eye GSS-PAK, and Red-eye GSS/Inv35-PAK strains. Significance symbols: “***” for *p* < 0.001, and “ns” for “not significant”.

There was no statistically significant difference between the PAK and Red-eye GSS-PAK strains in respect to these two traits (z = −1.98 (pupal), *p* = 0.1167 and z = −1.72 (adult), *p* = 0.199). However, significant reduction was observed in both pupal and adult recovery rates between the Red-eye GSS/Inv35 and the other two strains (PAK: z = −4.56 (pupal) and −4.18 (adult), both with *p* = 0.001, Red-eye GSS-PAK: z = −2.46 (pupal), *p* = 0.0367 and −2.34 (adult), *p* = 0.05). Statistically significant differences were also observed among the three strains in respect to the pupal and adult recovery rates of males and females of the PAK, Red-eye GSS-PAK, and Red-eye GSS/Inv35-PAK strains (Df = 1, F = 136.72, *p* = 8.83^−7^—Figure 5).

The average female and male pupal recovery rate were 35 and 37% for PAK, 23 and 42% for Red-eyes GSS-PAK, and 21 and 26% for Red-eyes GSS/Inv35-PAK, respectively. On the other hand, the average female and male adult recovery rate were 34 and 36% for PAK, 23 and 41% for Red-eyes GSS-PAK, and 21 and 26% for Red-eyes GSS/Inv35-PAK. Statistical analysis indicated significant difference in female pupal and adult recovery rates between PAK and Red-eye GSS-PAK (pupal: z = −2.98, *p* = 0.00787, and adult: z = −2.91, *p* = 0.00989), and between PAK and Red-eyes GSS/Inv35-PAK strain (pupal: z = −3.48, *p* = 0.00146, and adult: z = −3.44, *p* = 0.00166), but not between Red-eye GSS-PAK and Red-eyes GSS/Inv35-PAK (pupal: z = −0.55, *p* = 0.84643, and adult: z = −0.524, *p* = 0.85959) (Figure 5) In respect to the male pupal and adult recovery rates, the statistical analysis indicated no differences between PAK and Red-eyes GSS-PAK (pupal: z = 1.46, *p* = 0.30929, and adult: z = 1.54, *p* = 0.2745); however, there was difference between PAK and Red-eyes GSS/Inv35-PAK (pupal: z = −3.27, *p* = 0.00315, and adult: z = −2.86, *p* = 0.0119) as well as between Red-eyes GSS-PAK and Red-eyes GSS/Inv35-PAK (pupa: z = −4.62, *p* = 0.001, and adult: z = −4.28, *p* = 0.001) (Figure 5).

**FIGURE 6:**
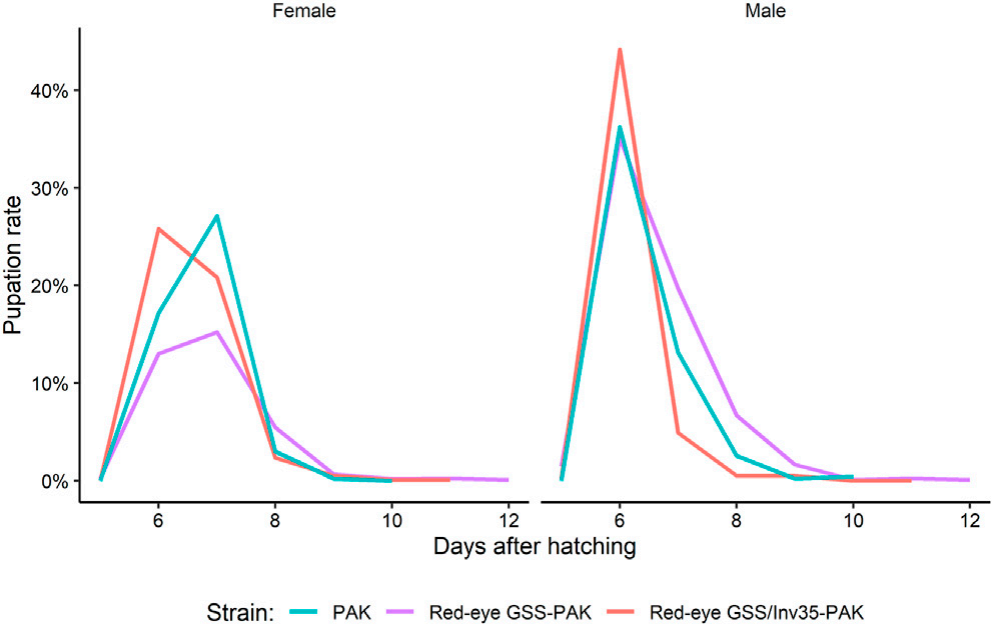
Pupation curve of males and females of the PAK, Red-eye GSS-PAK, and Red-eye GSS/Inv35-PAK strains.

There was no significant difference among the three strains on the pupation rate of males and females (Df = 1, F = 0.314, *p* = 0.57951). The maximum pupation rate for males was on the sixth day for all three strains. For females, the peak was observed on the seventh day for PAK and Red-eye GSS-PAK and on the sixth day for Red-eye GSS/Inv35-PAK (Figure 6).

#### Pupal Weight

**FIGURE 7:**
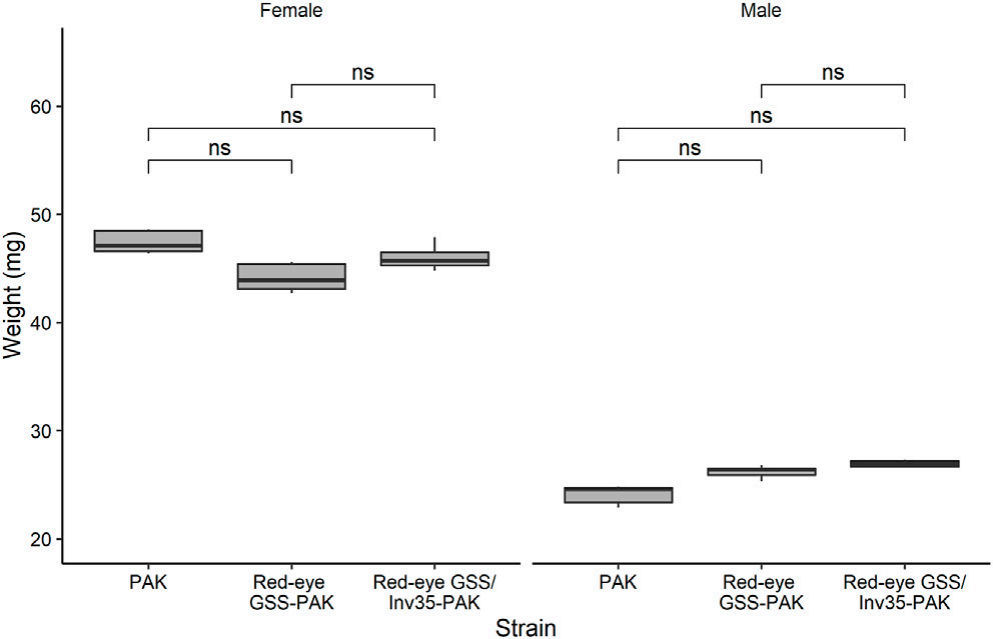
Pupal weight of males and females of the PAK, Red-eye GSS-PAK, and Red-eye GSS/Inv35-PAK strains. Significance symbol: “ns”—not significative.

No statistically significant differences were observed among the strains in respect to the weight (Df = 2, F = 1.776, *p* = 0.962) but, as expected, female pupae were heavier than male ones in all three strains studied (Df = 1, F = 1,194, *p* = 2^−16^—Figure 7).

#### Survival Rate

**FIGURE 8:**
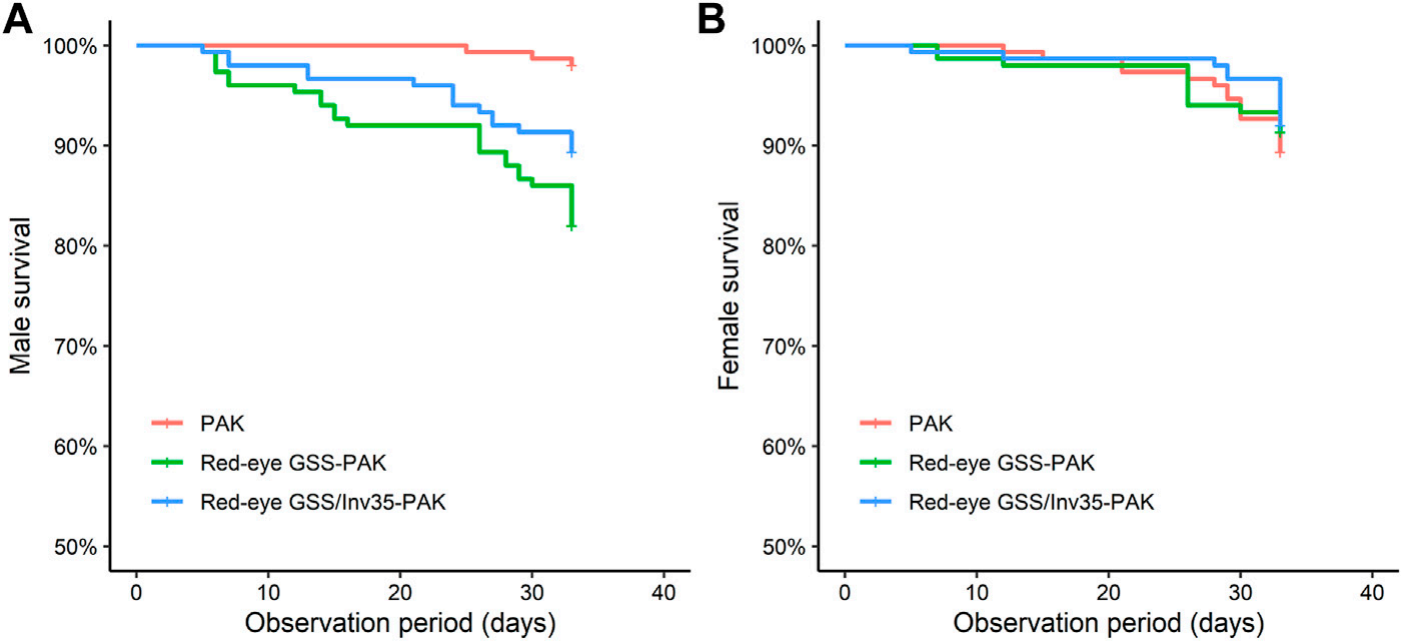
Survival rate of males **(A)** and females **(B)** of the PAK, Red-eye GSS-PAK, and Red-eye GSS/Inv35-PAK strains during the first 33 days post-emergence observation period.

Male survival rate of the Red-eye GSS-PAK and Red-eye GSS/Inv35-PAK strains was significantly reduced compared to PAK (Likelihood ratio test = 24.36, df = 2, *P* = 5^−6^—Figure 8A) while no statistically significant difference was observed in female survival rate (Likelihood ratio test = 0.7, df = 2, *p* = 0.7—Figure 8B) during the first 30 days period post emergence. It should be noted, however, that more than 85% of Red-eye GSS-PAK and Red-eye GSS/Inv35-PAK males were alive after the end of the observation period.

#### Flight Ability

**FIGURE 9:**
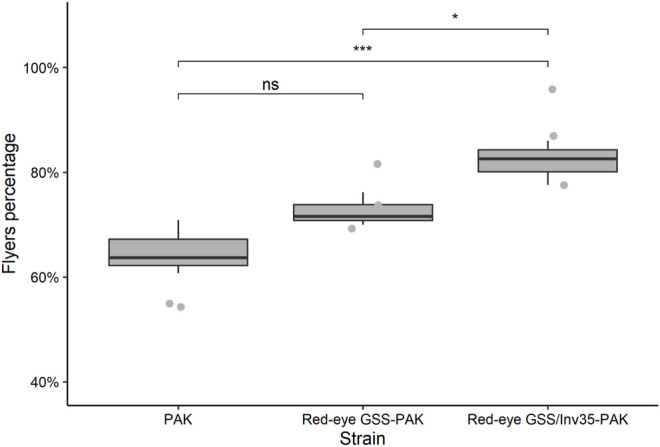
Flight ability of males of the PAK, Red-eye GSS-PAK, and Red-eye GSS/Inv35-PAK strains. Significance symbols: “***” for *p* < 0.001, “*” for *p* < 0.05, and “ns” for “not significant”.

In respect to the flight ability, the statistical analysis presented significant differences among the three strains (Df = 2, F = 11.74, *p* = 0.00844—Figure 9) with mean percentage of successful flyers being 65, 73 and 82% for the PAK, Red-eye GSS-PAK, and Red-eye GSS/Inv35-Pak strains. There was no difference between the PAK and Red-eye GSS-PAK strains (z = 2.04, *p* = 0.1016); however, there was significant difference between PAK and Red-eye GSS/Inv35-Pak (z = 4.78, *p* = 0.001) as well as between Red-eye GSS-PAK and Red-eye GSS/Inv35-Pak strains (z = 2.87, *p* = 0.0115).

## Discussion

Efficient, cost-effective, and safe SIT applications against major vector mosquito species, such as *Ae. aegypti* and *Ae. albopictus*, depend on efficient sex separation methods and the release of sterile males. The removal of *Aedes* female mosquitoes is needed because they bite, blood-feed and transmit pathogens such as chikungunya, dengue, yellow fever and ZIKA (Gilles et al., 2014; Papathanos et al., 2018; Lutrat et al., 2019). Current small-scale SIT trials are being carried out using local populations to minimize the risks associated with the introduction of vector mosquitoes of different origin (Bouyer et al., 2020; Carvalho et al., 2020; WHO and IAEA, 2020). Indeed, releasing GSS males carrying the local genomic background will enhance the efficiency of SIT since their mating competitiveness is likely to be higher than that of males of different origin. In addition, the SIT application with local males is not expected to raise biosafety and biosecurity concerns compared to a trial which would be based on mosquitoes originated from a different geographical region (Bouyer et al., 2020; WHO and IAEA, 2020; Augustinos et al., 2022). In the present study, the red eyes mutation and the inversion Inv35, which were used in the initial construction of the *Ae. aegypti* Red-eye GSS and Red-eye GSS/Inv35 strains (Augustinos et al., 2020; Koskinioti et al., 2021), were introduced into the genomic background of a wild-type PAK strain to assess their impact on their genetic stability, biological quality, and potential for SIT applications.

The genetic stability of GSS highly depends on recombination phenomena, which usually occur in males (Franz et al., 2021). Filtering systems and chromosomal inversions have been proposed as tools for the suppression of recombination and/or the removal of recombinants in order to maintain the genetic integrity of GSS (Franz et al., 2021). Unlike in fruit flies, genetic recombination occurs in both males and females of *Aedes* species, and this can significantly affect their stability, especially in the context of preserving the colony’s genetic integrity under mass rearing and female contamination in male-only releases for SIT programs (Augustinos et al., 2020; Franz et al., 2021). In our recently published studies, we reported the construction of the red-eye GSS (Koskinioti et al., 2021) and the introduction of Inv35 to suppress recombination (Augustinos et al., 2020). However, the recombination rate as well as the overall performance of a strain depends on several factors, including genomic background (Ouda and Wood, 1985; Augustinos et al., 2020, 2022; Carvalho et al., 2020). Therefore, in the present study, we introgressed the red eye mutation and the chromosomal inversion Inv35 in a wild population from Pakistan to assess the impact of the local genomic background on the genetic stability and the biological quality of newly constructed strains under laboratory rearing conditions.

As concerns the genetic stability, our results showed that genetic recombination was significantly suppressed in the presence of Inv35 and that the overall recombination rate in the Red-eye GSS-PAK and Red-eye GSS/Inv35-PAK strains was in the same range as described in the original strains, Red-eye GSS and Red-eye GSS/Inv35, reported in our previous study (Koskinioti et al., 2021). Taken together these data suggest that the genomic background did not have a significant impact on the genetic stability of the genetic sexing strains and are in accordance with recently reported recombination-suppressing properties of Inv35 (Augustinos et al., 2022).

One of the most important requirements for a successful SIT mosquito program is to mass produce and release high-quality sterile males that can compete with wild males for mating wild females (Bouyer and Vreysen, 2020; Parker et al., 2021). Quality of males is essential to determine the number of males to be released in the field, and high productivity, proper mating behavior, high survival, and good flight ability are among the desirable characters (Culbert et al., 2018; Parker et al., 2021). In the present study, we also determined the impact of the local Pakistani genomic background on the biological quality of the Red-eye GSS-PAK and the Red-eye GSS/Inv35-PAK under laboratory conditions by assessing parameters like fecundity, fertility, pupa and adult recovery, time of development, pupal weight, survival, and flight ability in comparison with the wild-type PAK strain.

Our results showed that the introgression had a positive impact on the fecundity of Red-eye GSS-PAK and Red-eye GSS/Inv35-PAK strains in both the first and the second gonotrophic cycle, similar to that reported for the originally constructed Red-eye GSS (Koskinioti et al., 2021). However, female pupal and adult recovery rate, and male survival rate were negatively affected. A positive impact on the flight ability of Red-eye GSS/Inv35-PAK males, compared to both PAK and Red-eye GSS-PAK males, was observed which could be attributed to heterozygote advantage. It is also important to note that the fertility as well as the male pupal and adult recovery rate was reduced in the Red-eye GSS/Inv35-PAK strain. On the other hand, the introgression had no effect on the pupation rate of males and females, and the pupal weight. The latter observation is very important in case a novel sex separation approach is developed based on both selectable markers, pupal size and eye color, as recently suggested (Koskinioti et al., 2021). In addition, it should be noted that, although the flight ability is a good indicator for the biological quality of males, proper evaluation of the male mating competitiveness of the Red-eye GSS and Red-eye GSS/Inv35 will be required prior to their use in any small- or large-scale field applications.

## Conclusion

Although the actual performance of a potential SIT strain can only be assessed in open-field conditions, laboratory characterization regarding genetic stability and biological quality is of utmost importance prior to mass production and releases of sterile males. Our present study studied biological traits or parameters, such as genetic recombination, fecundity, fertility, pupa and adult recovery, time of development, pupal weight, survival, and flight ability of two newly constructed introgressed strains Red-eye GSS-PAK and the Red-eye GSS/Inv35-PAK in comparison to the wild-type PAK strain. The results indicated that important biological quality parameters such as fecundity, fertility, pupal and adult recovery rate, survival rate, and flight ability, can be affected during the introgression process of different factors, such as the red-eye mutation and Inv35, into a new genomic background, which is in agreement with previous reports (Carvalho et al., 2020). Interestingly, some of these traits were affected in a sex specific manner. It is therefore recommended that the transfer of the selectable marker (red eye) and/or chromosomal inversion (Inv35) of the *Ae. aegypti* red eye GSS into new genomic backgrounds for the construction of the respective GSS should be accompanied by a thorough evaluation of the genetic stability and biological quality prior to its use in SIT applications in the field.

## Data Availability

The original contributions presented in the study are included in the article/Supplementary Material, further inquiries can be directed to the corresponding authors.
