# Supplementary material for: Genetic Stability and Fitness of Aedes aegypti Red-Eye Genetic Sexing Strains With Pakistani Genomic Background for Sterile Insect Technique Applications
Source: Front Bioeng Biotechnol. 2022 Mar 31;10:871703. doi: 10.3389/fbioe.2022.871703 (PMC9009520; doi:10.3389/fbioe.2022.871703)
Supplement: Supplementary file 1 [file DataSheet1.docx]

*Supplementary Material*

Acronyms used: RGSS for the Red-eye GSS-PAK strain and RGSS_35 for the Red-eye GSS/Inv35-PAK strain.

# Genetic Stability

Assessment of the recombination rate for each strain (Red-eye GSS-PAK and Red-eye GSS/Inv35-PAK) and sex over several generations.

**Table 01.** Recombination rate in each generation for each strain

| **Strain** | **Sex** | **Generation** | **Recombination rate (%)** |
| --- | --- | --- | --- |
| Red-eye GSS-PAK | Female | 1 | 1.399 |
|  |  | 2 | 2.012 |
|  |  | 3 | 2.385 |
|  |  | 4 | 4.463 |
|  |  | 6 | 1.808 |
|  |  | 7 | 1.987 |
|  | Male | 1 | 0.895 |
|  |  | 2 | 1.406 |
|  |  | 3 | 2.229 |
|  |  | 4 | 2.946 |
|  |  | 6 | 1.741 |
|  |  | 7 | 1.951 |
| Red-eye GSS/Inv35-PAK | Female | 1 | 0.278 |
|  |  | 2 | 0.108 |
|  |  | 3 | 0.702 |
|  |  | 4 | 0.597 |
|  |  | 6 | 0.952 |
|  |  | 7 | 0.605 |
|  | Male | 1 | 0.000 |
|  |  | 2 | 0.180 |
|  |  | 3 | 0.000 |
|  |  | 4 | 0.638 |
|  |  | 6 | 0.160 |
|  |  | 7 | 0.000 |

# Generalized Linear Model

GLM to indicate differences among the variables *strain*, in relation to the recombination rate. Strain GLM summary:

##
## Call: glm(formula = recombinants ~ strain, family = quasipoisson(link = "log"),
## data = recombinants_data %>% filter(sex == "female"))
##
## Coefficients:
## (Intercept) strainRGSS_35
## -3.754 -1.467
##
## Degrees of Freedom: 11 Total (i.e. Null); 10 Residual
## Null Deviance: 0.1047
## Residual Deviance: 0.03183 AIC: NA

##
## Call: glm(formula = recombinants ~ strain, family = quasipoisson(link = "log"),
## data = recombinants_data %>% filter(sex == "male"))
##
## Coefficients:
## (Intercept) strainRGSS_35
## -3.984 -2.436
##
## Degrees of Freedom: 11 Total (i.e. Null); 10 Residual
## Null Deviance: 0.1317
## Residual Deviance: 0.03131 AIC: NA

Comparison between the groups:

| **Sex** | **group1** | **group2** | **P value** | **signif** |
| --- | --- | --- | --- | --- |
| female | RGSS_35 | RGSS | 4.16e-05 | *** |
| male | RGSS_35 | RGSS | 4.53e-05 | *** |


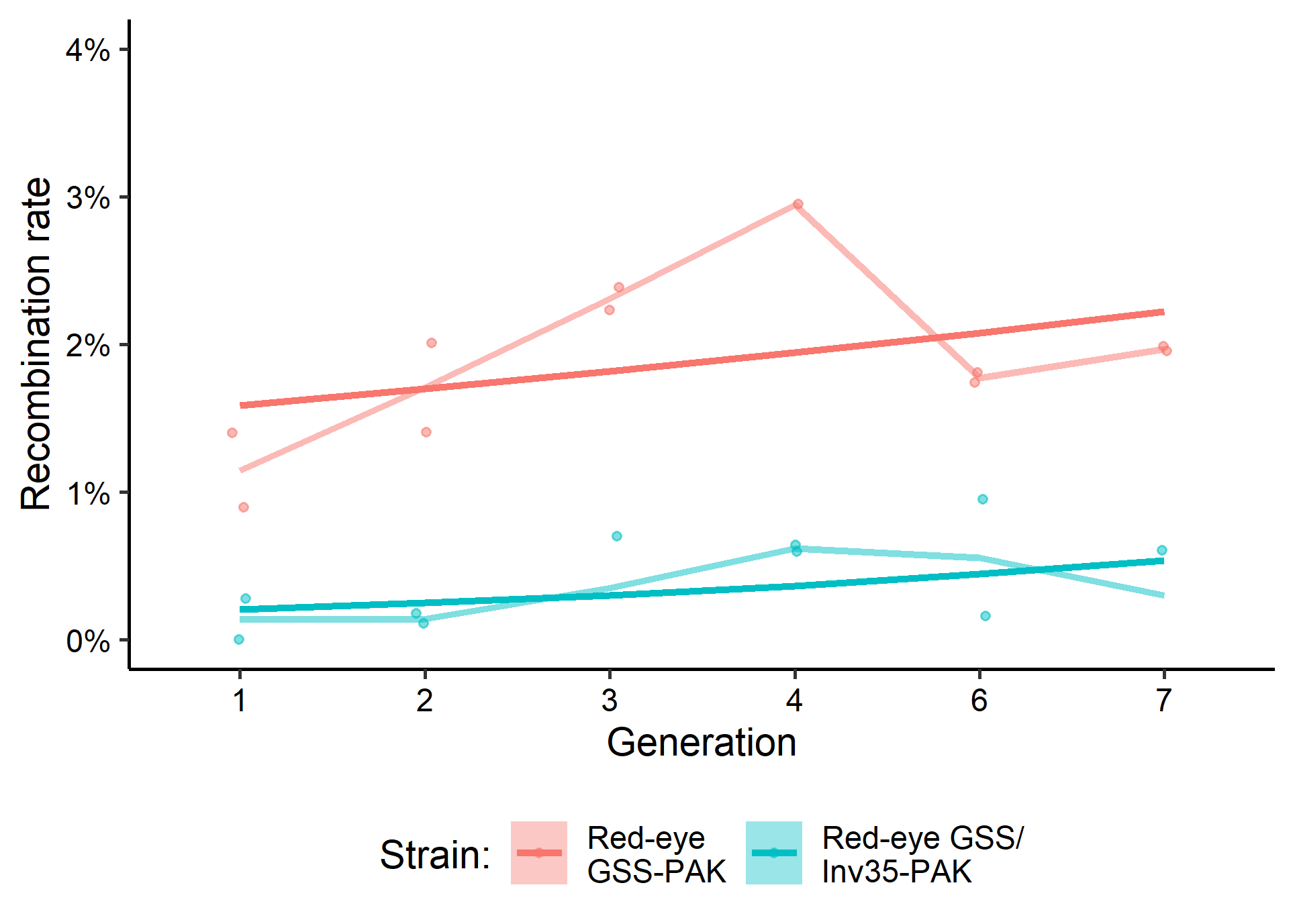


**Figure 1.** Recombination rate in Red-eye GSS-PAK and Red-eye GSS/Inv35-PAK strains during seven generations.

# Fecundity

The fecundity was defined as the average number of eggs per female for each strain.

**Table 03.** Average number of eggs/female in each gonotrophic cycle for each strain.

| **Strain** | **Gonotrophic Cycle** | **Eggs/female** | **Standard Error** |
| --- | --- | --- | --- |
| PAK | 1 | 31.97 | 3.87 |
|  | 2 | 21.60 | 1.22 |
| Red-eye GSS-PAK | 1 | 68.60 | 1.70 |
|  | 2 | 55.67 | 8.77 |
| Red-eye GSS/Inv35-PAK | 1 | 74.27 | 7.11 |
|  | 2 | 60.80 | 9.43 |

# Generalized Linear Model

##
## Call: glm(formula = n_eggs/n_females ~ strain * GC * rep, family = poisson(link = "log"),
## data = fecundity, weights = n_females)
##
## Coefficients:
## (Intercept) strainRGSS strainRGSS_35
## 3.40811 0.90416 0.74670
## GC2 rep strainRGSS:GC2
## -0.22528 0.02816 0.31021
## strainRGSS_35:GC2 strainRGSS:rep strainRGSS_35:rep
## -0.12817 -0.07044 0.04732
## GC2:rep strainRGSS:GC2:rep strainRGSS_35:GC2:rep
## -0.08374 -0.06921 0.15767
##
## Degrees of Freedom: 17 Total (i.e. Null); 6 Residual
## Null Deviance: 1653
## Residual Deviance: 170.6 AIC: Inf

## Df Sum Sq Mean Sq F value Pr(>F)
## strain 2 58787 29393 18.897 0.00257 **
## GC 1 6759 6759 4.345 0.08222 .
## rep 1 1 1 0.001 0.97697
## strain:GC 2 82 41 0.026 0.97397
## strain:rep 2 4023 2011 1.293 0.34124
## GC:rep 1 143 143 0.092 0.77196
## strain:GC:rep 2 644 322 0.207 0.81853
## Residuals 6 9333 1555
## ---
## Signif. codes: 0 '***' 0.001 '**' 0.01 '*' 0.05 '.' 0.1 ' ' 1

##
## Simultaneous Tests for General Linear Hypotheses
##
## Multiple Comparisons of Means: Tukey Contrasts
##
##
## Fit: glm(formula = n_eggs/n_females ~ strain * GC * rep, family = poisson(link = "log"),
## data = fecundity, weights = n_females)
##
## Linear Hypotheses:
## Estimate Std. Error z value Pr(>|z|)
## RGSS - PAK == 0 0.9042 0.1036 8.724 <1e-04 ***
## RGSS_35 - PAK == 0 0.7467 0.1035 7.216 <1e-04 ***
## RGSS_35 - RGSS == 0 -0.1575 0.0813 -1.937 0.127
## ---
## Signif. codes: 0 '***' 0.001 '**' 0.01 '*' 0.05 '.' 0.1 ' ' 1
## (Adjusted p values reported -- single-step method)


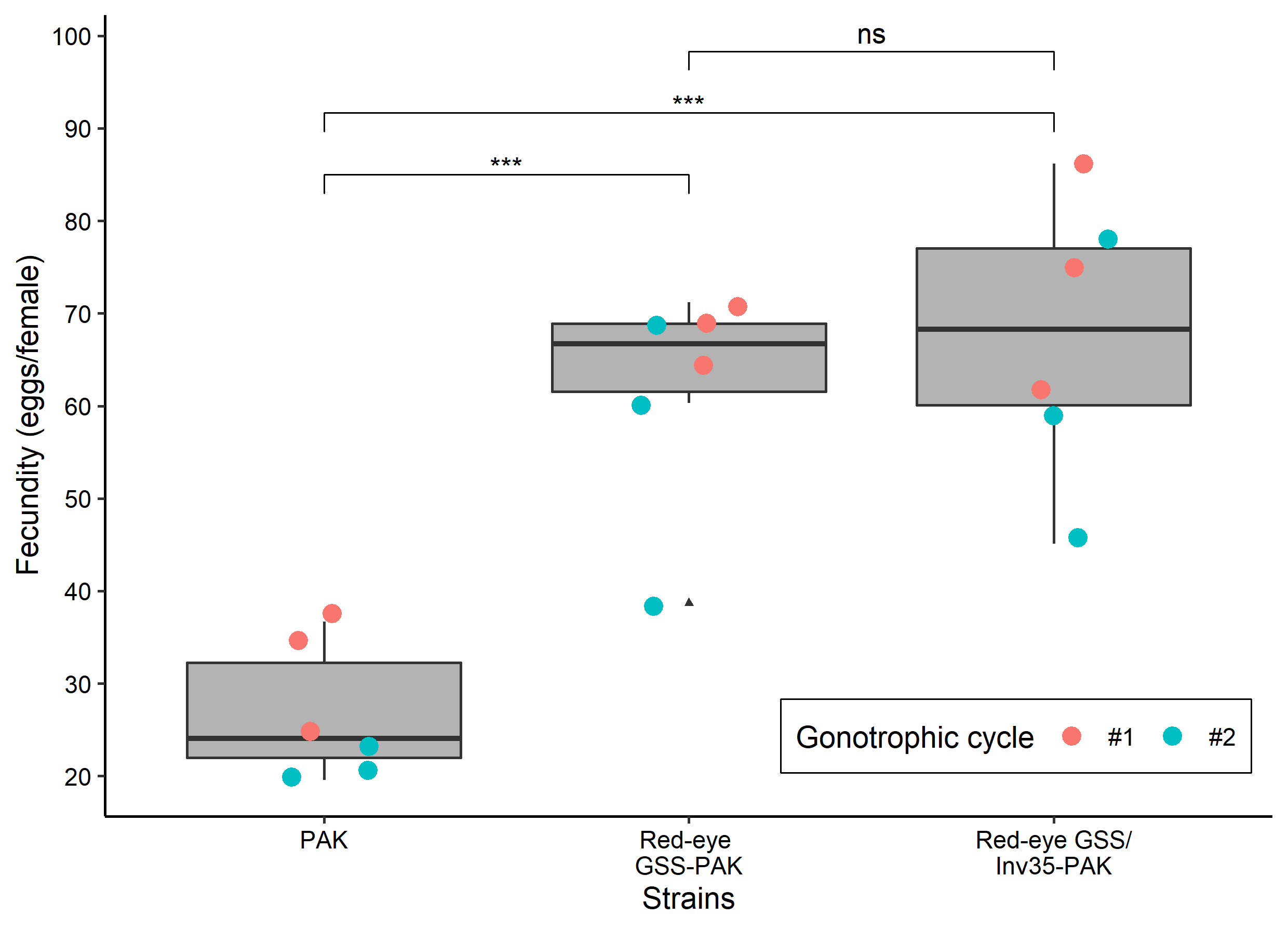


**Figure 2.** Fecundity of the PAK, Red-eye GSS-PAK, and Red-eye GSS/Inv35-PAK strains during the first and second gonotrophic cycle.

# Fertility

The fertility was defined as the mean egg hatch rate for each strain in each gonotrophic cycle.

**Table 04.** Mean egg hatch rate (%) in each gonotrophic cycle for each strain

| **Strain** | **Gonotrophic cycle** | **Hatch rate (%)** | **Standard error** |
| --- | --- | --- | --- |
| PAK | 1 | 83.01 | 1.18 |
|  | 2 | 67.46 | 3.96 |
| Red-eye GSS-PAK | 1 | 71.14 | 11.88 |
|  | 2 | 70.71 | 8.55 |
| Red-eye GSS/Inv35-PAK | 1 | 46.72 | 12.08 |
|  | 2 | 49.36 | 2.98 |

# Generalized Linear Model

##
## Call: glm(formula = n_larvae/n_eggs ~ strain, family = quasibinomial(link = "logit"),
## data = fertility, weights = n_larvae)
##
## Coefficients:
## (Intercept) strainRGSS strainRGSS_35
## 1.2660 -0.1806 -1.1643
##
## Degrees of Freedom: 17 Total (i.e. Null); 15 Residual
## Null Deviance: 668.8
## Residual Deviance: 368.3 AIC: NA

## Df Sum Sq Mean Sq F value Pr(>F)
## strain 2 67.25 33.62 6.779 0.00799 **
## Residuals 15 74.40 4.96
## ---
## Signif. codes: 0 '***' 0.001 '**' 0.01 '*' 0.05 '.' 0.1 ' ' 1

##
## Simultaneous Tests for General Linear Hypotheses
##
## Multiple Comparisons of Means: Tukey Contrasts
##
##
## Fit: glm(formula = n_larvae/n_eggs ~ strain, family = quasibinomial(link = "logit"),
## data = fertility, weights = n_larvae)
##
## Linear Hypotheses:
## Estimate Std. Error z value Pr(>|z|)
## RGSS - PAK == 0 -0.1806 0.4885 -0.370 0.92584
## RGSS_35 - PAK == 0 -1.1643 0.4890 -2.381 0.04370 *
## RGSS_35 - RGSS == 0 -0.9837 0.3150 -3.123 0.00485 **
## ---
## Signif. codes: 0 '***' 0.001 '**' 0.01 '*' 0.05 '.' 0.1 ' ' 1
## (Adjusted p values reported -- single-step method)


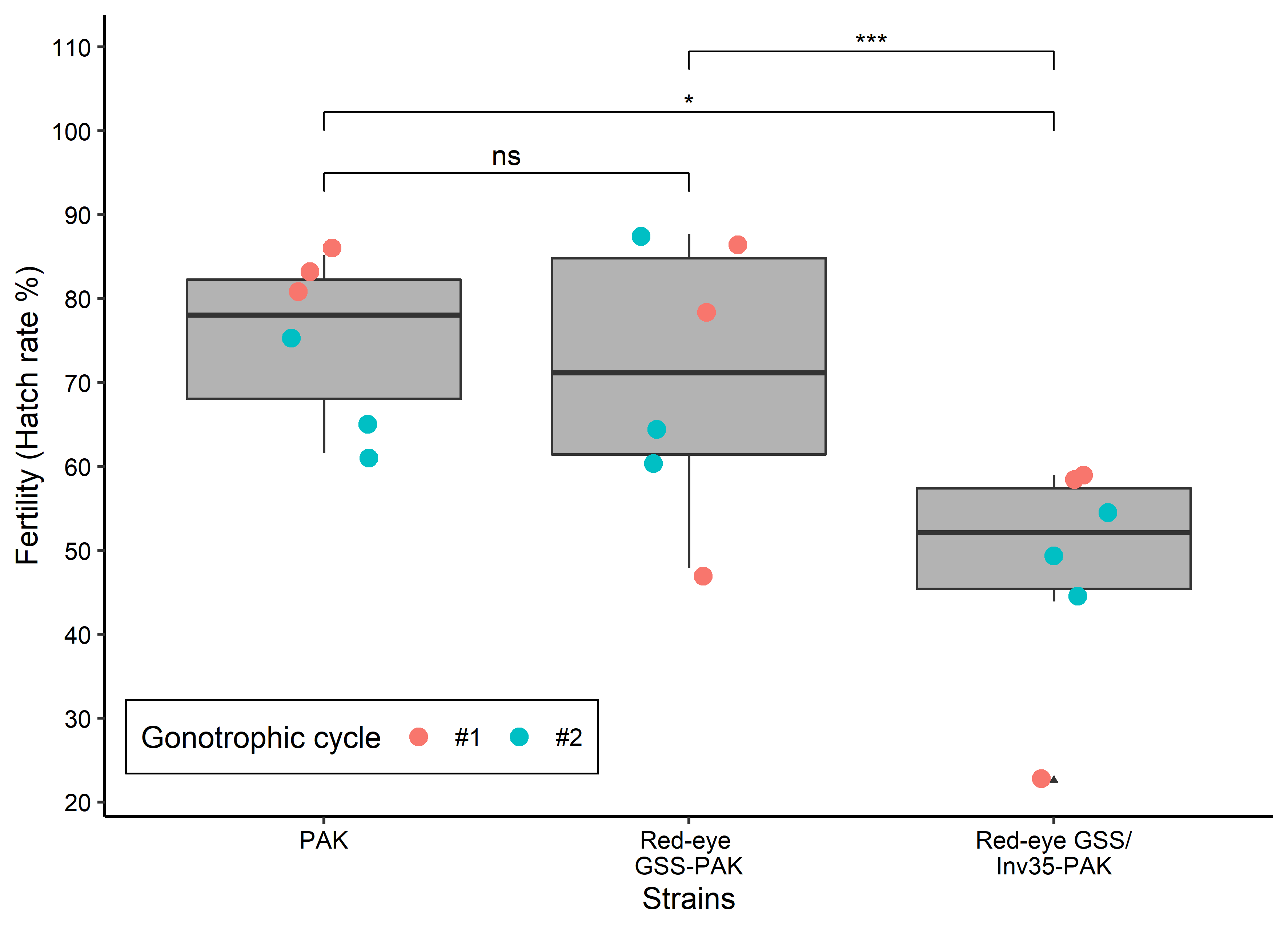


**Figure 3.** Fertility of the PAK, Red-eye GSS-PAK, and Red-eye GSS/Inv35-PAK strains.

# Pupal and adult recovery rates

Recovery was defined as the percentage of individuals reaching a desired stage (pupal or adult), from a known initial number of individuals.

**Table 05.** Pupal and adult recovery rates for Red-eye GSS-PAK, Red-eye GSS/Inv35-PAK, and PAK strains.

| **Strain** | **Stage** | **Average recovery** | **Standard error** |
| --- | --- | --- | --- |
| PAK | pupal | 0.72 | 0.03 |
|  | adult | 0.70 | 0.04 |
| Red-eye GSS-PAK | pupal | 0.60 | 0.04 |
|  | adult | 0.59 | 0.04 |
| Red-eye GSS/Inv35-PAK | pupal | 0.45 | 0.05 |
|  | adult | 0.44 | 0.05 |

# Generalized Linear Model - Overall

##
## Call: glm(formula = recovery ~ strain, family = quasibinomial(link = "logit"),
## data = rec_pupa_adult %>% filter(sex == "both", stage ==
## "pupa"))
##
## Coefficients:
## (Intercept) strainRGSS strainRGSS_INV35
## 0.9473 -0.5293 -1.1564
##
## Degrees of Freedom: 16 Total (i.e. Null); 14 Residual
## Null Deviance: 1.553
## Residual Deviance: 0.6163 AIC: NA

## Df Sum Sq Mean Sq F value Pr(>F)
## strain 2 0.2243 0.11214 11.18 0.00125 **
## Residuals 14 0.1404 0.01003
## ---
## Signif. codes: 0 '***' 0.001 '**' 0.01 '*' 0.05 '.' 0.1 ' ' 1

##
## Simultaneous Tests for General Linear Hypotheses
##
## Multiple Comparisons of Means: Tukey Contrasts
##
##
## Fit: glm(formula = recovery ~ strain, family = quasibinomial(link = "logit"),
## data = rec_pupa_adult %>% filter(sex == "both", stage ==
## "pupa"))
##
## Linear Hypotheses:
## Estimate Std. Error z value Pr(>|z|)
## RGSS - WILD == 0 -0.5293 0.2671 -1.981 0.1167
## RGSS_INV35 - WILD == 0 -1.1564 0.2538 -4.556 <0.001 ***
## RGSS_INV35 - RGSS == 0 -0.6271 0.2545 -2.464 0.0366 *
## ---
## Signif. codes: 0 '***' 0.001 '**' 0.01 '*' 0.05 '.' 0.1 ' ' 1
## (Adjusted p values reported -- single-step method)

##
## Call: glm(formula = recovery ~ strain, family = quasibinomial(link = "logit"),
## data = rec_pupa_adult %>% filter(sex == "both", stage ==
## "adult"))
##
## Coefficients:
## (Intercept) strainRGSS strainRGSS_INV35
## 0.8414 -0.4667 -1.0808
##
## Degrees of Freedom: 16 Total (i.e. Null); 14 Residual
## Null Deviance: 1.489
## Residual Deviance: 0.654 AIC: NA

## Df Sum Sq Mean Sq F value Pr(>F)
## strain 2 0.2021 0.10103 9.434 0.00254 **
## Residuals 14 0.1499 0.01071
## ---
## Signif. codes: 0 '***' 0.001 '**' 0.01 '*' 0.05 '.' 0.1 ' ' 1

##
## Simultaneous Tests for General Linear Hypotheses
##
## Multiple Comparisons of Means: Tukey Contrasts
##
##
## Fit: glm(formula = recovery ~ strain, family = quasibinomial(link = "logit"),
## data = rec_pupa_adult %>% filter(sex == "both", stage ==
## "adult"))
##
## Linear Hypotheses:
## Estimate Std. Error z value Pr(>|z|)
## RGSS - WILD == 0 -0.4667 0.2719 -1.716 0.1988
## RGSS_INV35 - WILD == 0 -1.0808 0.2588 -4.176 <0.001 ***
## RGSS_INV35 - RGSS == 0 -0.6141 0.2620 -2.344 0.0501 .
## ---
## Signif. codes: 0 '***' 0.001 '**' 0.01 '*' 0.05 '.' 0.1 ' ' 1
## (Adjusted p values reported -- single-step method)


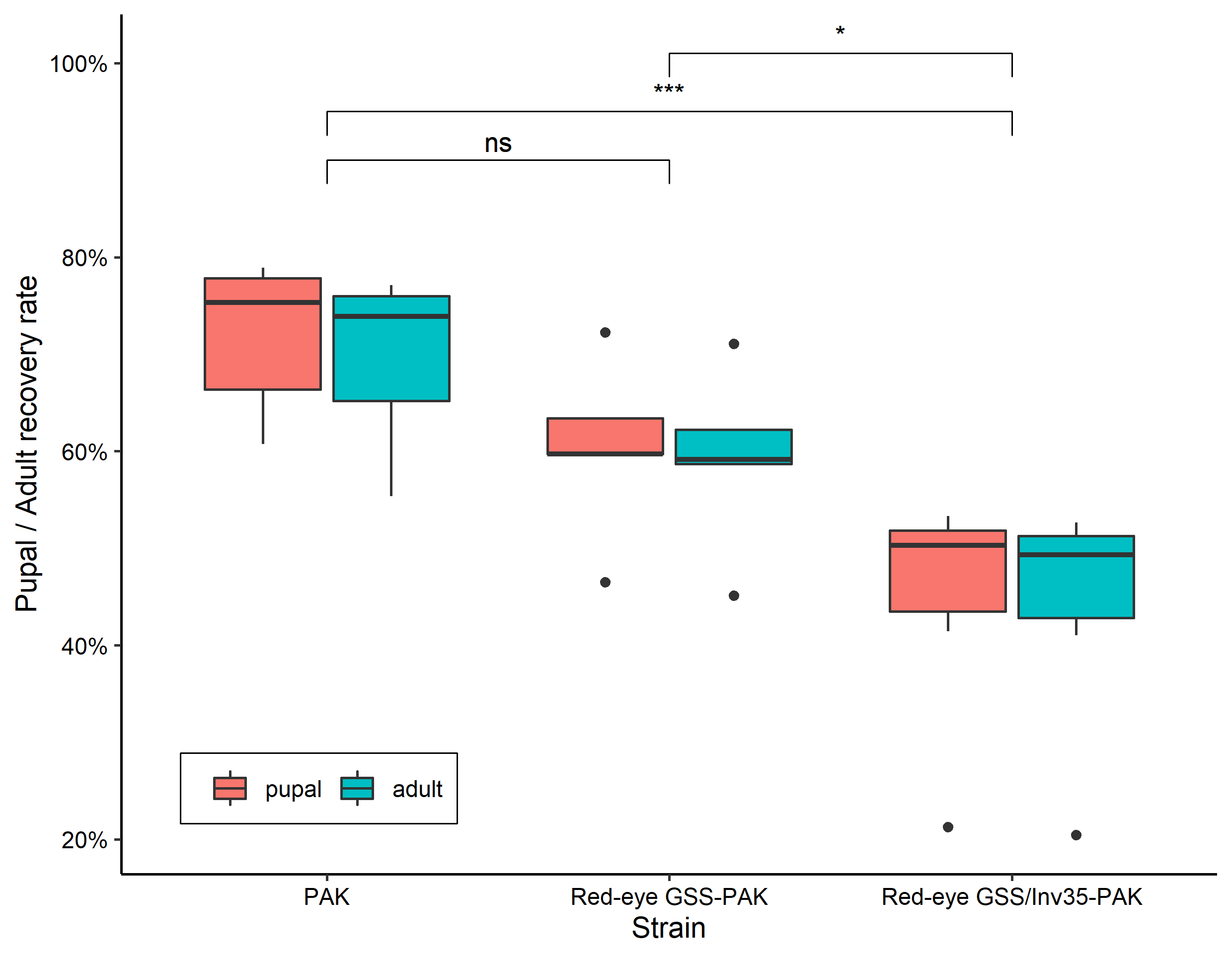


**Figure 4.** Pupal and adult recovery rate of the PAK, Red-eye GSS-PAK, and Red-eye GSS/Inv35-PAK strains.

**Table 06.** Pupal and adult recovery rate of males and females of the PAK, Red-eye GSS-PAK, and Red-eye GSS/Inv35-PAK strains

| **Strain** | **Sex** | **Average Recovery** | **Standard Error** |
| --- | --- | --- | --- |
| PAK | female | 0.35 | 0.02 |
|  | male | 0.37 | 0.02 |
| Red-eye GSS-PAK | female | 0.23 | 0.04 |
|  | male | 0.42 | 0.03 |
| Red-eye GSS/Inv35-PAK | female | 0.21 | 0.02 |
|  | male | 0.26 | 0.02 |

# Generalized Linear Model - Sex

##
## Call: glm(formula = recovery ~ strain, family = quasibinomial(link = "logit"),
## data = rec_pupa_adult %>% filter(stage == "pupa", sex ==
## "female"))
##
## Coefficients:
## (Intercept) strainRGSS strainRGSS_INV35
## -0.6175 -0.5845 -0.6950
##
## Degrees of Freedom: 17 Total (i.e. Null); 15 Residual
## Null Deviance: 0.6932
## Residual Deviance: 0.3548 AIC: NA

## Df Sum Sq Mean Sq F value Pr(>F)
## strain 2 0.06740 0.03370 8.15 0.00402 **
## Residuals 15 0.06202 0.00413
## ---
## Signif. codes: 0 '***' 0.001 '**' 0.01 '*' 0.05 '.' 0.1 ' ' 1

##
## Simultaneous Tests for General Linear Hypotheses
##
## Multiple Comparisons of Means: Tukey Contrasts
##
##
## Fit: glm(formula = recovery ~ strain, family = quasibinomial(link = "logit"),
## data = rec_pupa_adult %>% filter(stage == "pupa", sex ==
## "female"))
##
## Linear Hypotheses:
## Estimate Std. Error z value Pr(>|z|)
## RGSS - WILD == 0 -0.5845 0.1959 -2.983 0.00805 **
## RGSS_INV35 - WILD == 0 -0.6950 0.1994 -3.485 0.00140 **
## RGSS_INV35 - RGSS == 0 -0.1105 0.2109 -0.524 0.85959
## ---
## Signif. codes: 0 '***' 0.001 '**' 0.01 '*' 0.05 '.' 0.1 ' ' 1
## (Adjusted p values reported -- single-step method)

##
## Call: glm(formula = recovery ~ strain, family = quasibinomial(link = "logit"),
## data = rec_pupa_adult %>% filter(stage == "pupa", sex ==
## "male"))
##
## Coefficients:
## (Intercept) strainRGSS strainRGSS_INV35
## -0.5313 0.1963 -0.4898
##
## Degrees of Freedom: 16 Total (i.e. Null); 14 Residual
## Null Deviance: 0.4728
## Residual Deviance: 0.1822 AIC: NA

## Df Sum Sq Mean Sq F value Pr(>F)
## strain 2 0.06515 0.03257 10.8 0.00145 **
## Residuals 14 0.04221 0.00301
## ---
## Signif. codes: 0 '***' 0.001 '**' 0.01 '*' 0.05 '.' 0.1 ' ' 1

##
## Simultaneous Tests for General Linear Hypotheses
##
## Multiple Comparisons of Means: Tukey Contrasts
##
##
## Fit: glm(formula = recovery ~ strain, family = quasibinomial(link = "logit"),
## data = rec_pupa_adult %>% filter(stage == "pupa", sex ==
## "male"))
##
## Linear Hypotheses:
## Estimate Std. Error z value Pr(>|z|)
## RGSS - WILD == 0 0.1963 0.1344 1.461 0.30929
## RGSS_INV35 - WILD == 0 -0.4898 0.1499 -3.268 0.00307 **
## RGSS_INV35 - RGSS == 0 -0.6862 0.1486 -4.617 < 0.001 ***
## ---
## Signif. codes: 0 '***' 0.001 '**' 0.01 '*' 0.05 '.' 0.1 ' ' 1
## (Adjusted p values reported -- single-step method)

##
## Call: glm(formula = recovery ~ strain, family = quasibinomial(link = "logit"),
## data = rec_pupa_adult %>% filter(stage != "pupa", sex ==
## "female"))
##
## Coefficients:
## (Intercept) strainRGSS strainRGSS_INV35
## -0.6555 -0.5651 -0.6799
##
## Degrees of Freedom: 17 Total (i.e. Null); 15 Residual
## Null Deviance: 0.6615
## Residual Deviance: 0.3443 AIC: NA

## Df Sum Sq Mean Sq F value Pr(>F)
## strain 2 0.06235 0.031173 7.883 0.00457 **
## Residuals 15 0.05932 0.003954
## ---
## Signif. codes: 0 '***' 0.001 '**' 0.01 '*' 0.05 '.' 0.1 ' ' 1

##
## Simultaneous Tests for General Linear Hypotheses
##
## Multiple Comparisons of Means: Tukey Contrasts
##
##
## Fit: glm(formula = recovery ~ strain, family = quasibinomial(link = "logit"),
## data = rec_pupa_adult %>% filter(stage != "pupa", sex ==
## "female"))
##
## Linear Hypotheses:
## Estimate Std. Error z value Pr(>|z|)
## RGSS - WILD == 0 -0.5651 0.1939 -2.915 0.00997 **
## RGSS_INV35 - WILD == 0 -0.6799 0.1975 -3.443 0.00189 **
## RGSS_INV35 - RGSS == 0 -0.1148 0.2088 -0.550 0.84643
## ---
## Signif. codes: 0 '***' 0.001 '**' 0.01 '*' 0.05 '.' 0.1 ' ' 1
## (Adjusted p values reported -- single-step method)

##
## Call: glm(formula = recovery ~ strain, family = quasibinomial(link = "logit"),
## data = rec_pupa_adult %>% filter(stage != "pupa", sex ==
## "male"))
##
## Coefficients:
## (Intercept) strainRGSS strainRGSS_INV35
## -0.5884 0.2175 -0.4521
##
## Degrees of Freedom: 16 Total (i.e. Null); 14 Residual
## Null Deviance: 0.4726
## Residual Deviance: 0.202 AIC: NA

## Df Sum Sq Mean Sq F value Pr(>F)
## strain 2 0.06011 0.030053 9.139 0.00289 **
## Residuals 14 0.04604 0.003288
## ---
## Signif. codes: 0 '***' 0.001 '**' 0.01 '*' 0.05 '.' 0.1 ' ' 1

##
## Simultaneous Tests for General Linear Hypotheses
##
## Multiple Comparisons of Means: Tukey Contrasts
##
##
## Fit: glm(formula = recovery ~ strain, family = quasibinomial(link = "logit"),
## data = rec_pupa_adult %>% filter(stage != "pupa", sex ==
## "male"))
##
## Linear Hypotheses:
## Estimate Std. Error z value Pr(>|z|)
## RGSS - WILD == 0 0.2175 0.1418 1.534 0.2746
## RGSS_INV35 - WILD == 0 -0.4521 0.1582 -2.857 0.0118 *
## RGSS_INV35 - RGSS == 0 -0.6696 0.1566 -4.276 <0.001 ***
## ---
## Signif. codes: 0 '***' 0.001 '**' 0.01 '*' 0.05 '.' 0.1 ' ' 1
## (Adjusted p values reported -- single-step method)


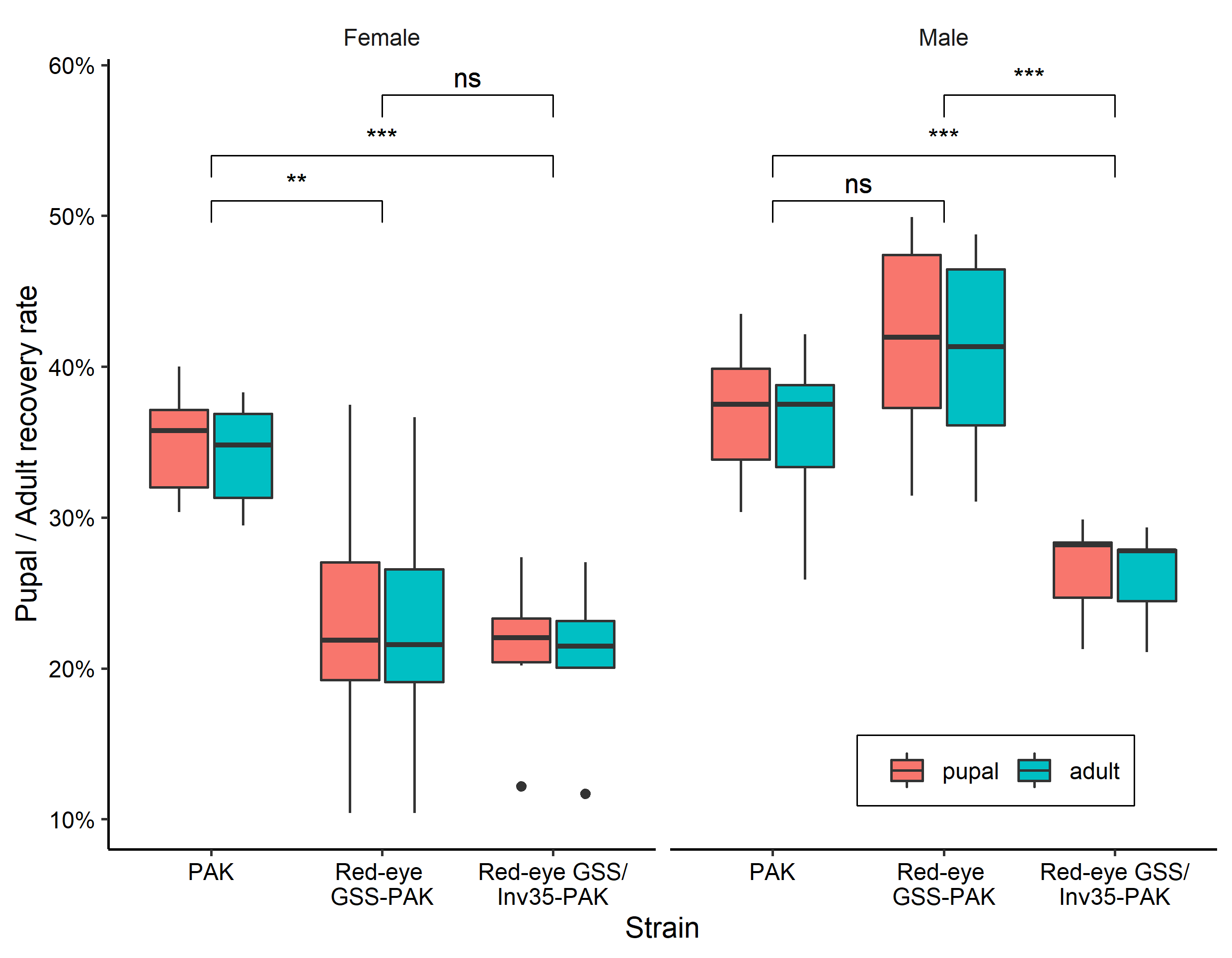


**Figure 5.** Pupal and adult recovery rate of males and females of the PAK, Red-eye GSS-PAK, and Red-eye GSS/Inv35-PAK strains.

# Pupation curve

Pupation curve was defined as the number of pupae collected/day since egg hatching.

# Generalized Linear Model

##
## Call: glm(formula = countings ~ strain * sex * day, data = pupation_data)
##
## Coefficients:
## (Intercept) strainRGSS_35
## 196.857 72.714
## strainPAK sexmale
## -83.667 210.917
## day strainRGSS_35:sexmale
## -16.821 -148.631
## strainPAK:sexmale strainRGSS_35:day
## -164.917 -8.179
## strainPAK:day sexmale:day
## 6.707 -19.417
## strainRGSS_35:sexmale:day strainPAK:sexmale:day
## 11.738 13.817
##
## Degrees of Freedom: 41 Total (i.e. Null); 30 Residual
## Null Deviance: 498200
## Residual Deviance: 358700 AIC: 525.4

## Df Sum Sq Mean Sq F value Pr(>F)
## strain 2 10431 5215 0.436 0.65057
## sex 1 3753 3753 0.314 0.57951
## day 1 105436 105436 8.817 0.00582 **
## strain:sex 2 4716 2358 0.197 0.82209
## strain:day 2 6098 3049 0.255 0.77660
## sex:day 1 7277 7277 0.609 0.44144
## strain:sex:day 2 1740 870 0.073 0.93000
## Residuals 30 358745 11958
## ---
## Signif. codes: 0 '***' 0.001 '**' 0.01 '*' 0.05 '.' 0.1 ' ' 1


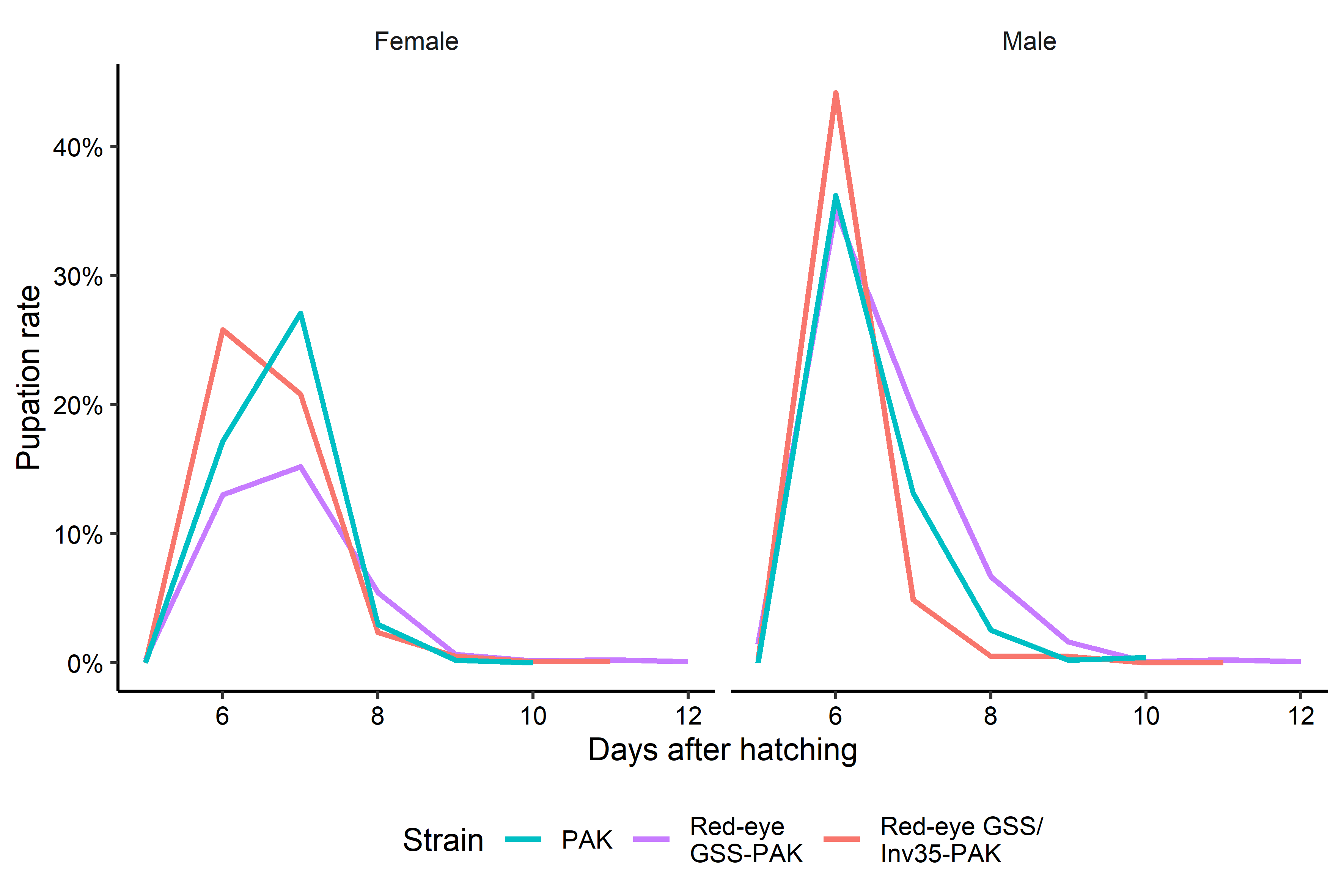


**Figure 6.** Pupation curve of males and females of the PAK, Red-eye GSS-PAK, and Red-eye GSS/Inv35-PAK strains.

# Pupal weight

The mean pupal weight obtained from 10 pupae for each sex and strain.

**Table 07.** Mean pupal weight (and standard error) of males and females of the PAK, Red-eye GSS-PAK, and Red-eye GSS/Inv35-PAK strains

| **Strain** | **Sex** | **Weight (mg)** | **Standard error** |
| --- | --- | --- | --- |
| Red-eye GSS-PAK | female | 47.44 | 0.468 |
|  | male | 24.08 | 0.389 |
| Red-eye GSS/Inv35-PAK | female | 44.14 | 0.589 |
|  | male | 26.18 | 0.263 |
| PAK | female | 46.04 | 0.542 |
|  | male | 26.94 | 0.136 |

# Generalized Linear Model

##
## Call: glm(formula = weight ~ strain, family = poisson(link = "log"),
## data = weight %>% filter(sex == "female"))
##
## Coefficients:
## (Intercept) strain.L strain.Q
## -3.08231 -0.02118 0.04664
##
## Degrees of Freedom: 14 Total (i.e. Null); 12 Residual
## Null Deviance: 0.0009751
## Residual Deviance: 0.0003758 AIC: Inf

## Df Sum Sq Mean Sq F value Pr(>F)
## strain 2 2.743e-05 1.372e-05 9.583 0.00326 **
## Residuals 12 1.718e-05 1.431e-06
## ---
## Signif. codes: 0 '***' 0.001 '**' 0.01 '*' 0.05 '.' 0.1 ' ' 1

##
## Simultaneous Tests for General Linear Hypotheses
##
## Multiple Comparisons of Means: Tukey Contrasts
##
##
## Fit: glm(formula = weight ~ strain, family = poisson(link = "log"),
## data = weight %>% filter(sex == "female"))
##
## Linear Hypotheses:
## Estimate Std. Error z value Pr(>|z|)
## RGSS - PAK == 0 -0.07210 2.95750 -0.024 1
## RGSS_35 - PAK == 0 -0.02996 2.92572 -0.010 1
## RGSS_35 - RGSS == 0 0.04214 2.97909 0.014 1
## (Adjusted p values reported -- single-step method)

##
## Call: glm(formula = weight ~ strain, family = poisson(link = "log"),
## data = weight %>% filter(sex == "male"))
##
## Coefficients:
## (Intercept) strain.L strain.Q
## -3.66109 0.07936 -0.02245
##
## Degrees of Freedom: 14 Total (i.e. Null); 12 Residual
## Null Deviance: 0.001054
## Residual Deviance: 0.0001935 AIC: Inf

## Df Sum Sq Mean Sq F value Pr(>F)
## strain 2 2.195e-05 1.097e-05 27.5 3.3e-05 ***
## Residuals 12 4.788e-06 3.990e-07
## ---
## Signif. codes: 0 '***' 0.001 '**' 0.01 '*' 0.05 '.' 0.1 ' ' 1

##
## Simultaneous Tests for General Linear Hypotheses
##
## Multiple Comparisons of Means: Tukey Contrasts
##
##
## Fit: glm(formula = weight ~ strain, family = poisson(link = "log"),
## data = weight %>% filter(sex == "male"))
##
## Linear Hypotheses:
## Estimate Std. Error z value Pr(>|z|)
## RGSS - PAK == 0 0.08361 3.99313 0.021 1
## RGSS_35 - PAK == 0 0.11223 3.96605 0.028 1
## RGSS_35 - RGSS == 0 0.02862 3.88115 0.007 1
## (Adjusted p values reported -- single-step method)


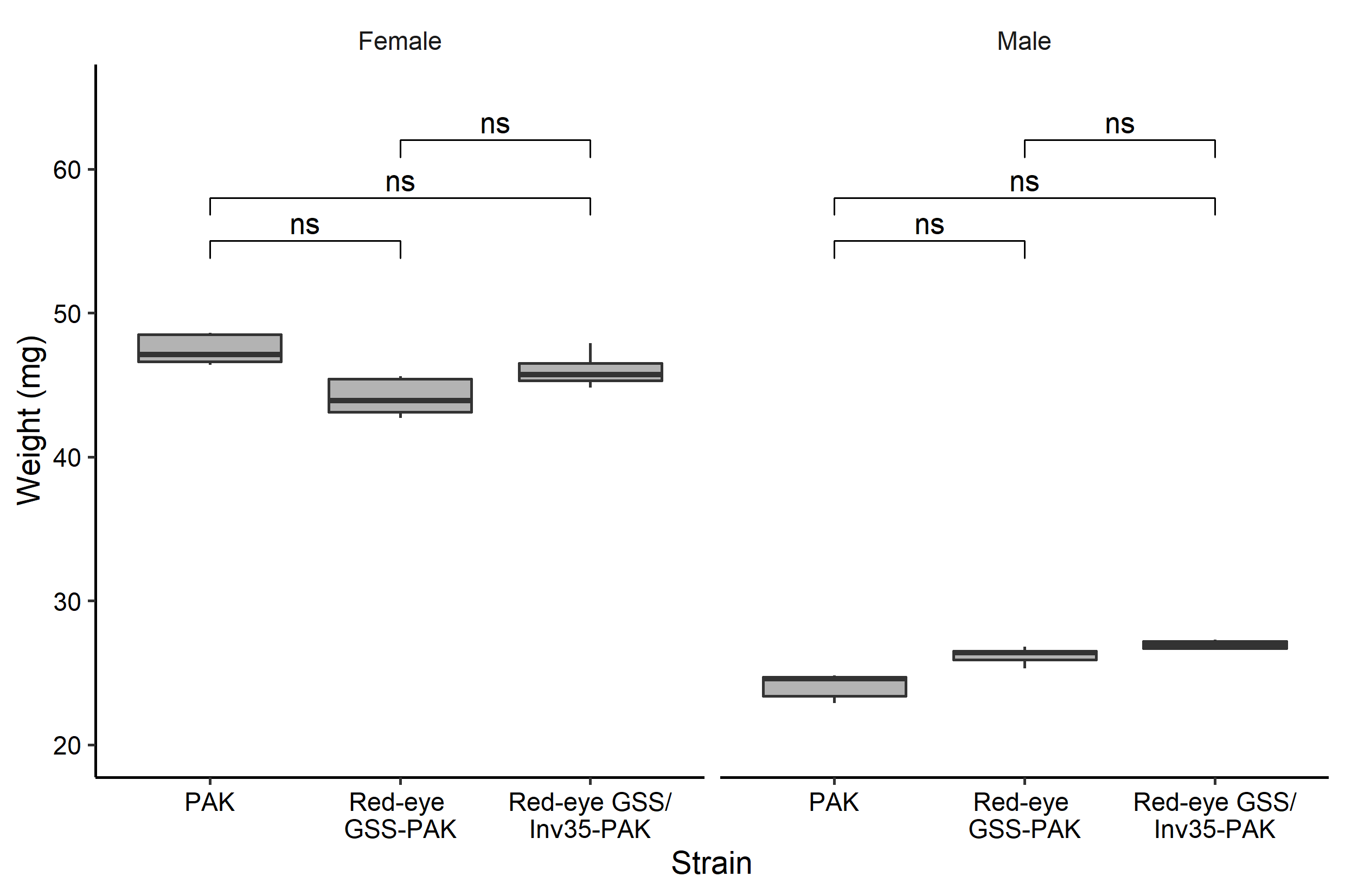


**Figure 7.** Pupal weight of males and females of the PAK, Red-eye GSS-PAK, and Red-eye GSS/Inv35-PAK strains.

# Survival rate

# Generalized Linear Model - Males

## Call:
## survdiff(formula = Surv(time, status) ~ strain, data = male_survival_curve)
##
## N Observed Expected (O-E)^2/E (O-E)^2/V
## strain=WILD 150 3 16.0 10.5710 16.3493
## strain=RGSS 150 27 14.7 10.1963 15.1272
## strain=RGSS_35 150 16 15.3 0.0368 0.0555
##
## Chisq= 21 on 2 degrees of freedom, p= 3e-05

## Call:
## coxph(formula = Surv(time, status) ~ strain, data = male_survival_curve)
##
## n= 450, number of events= 46
##
## coef exp(coef) se(coef) z Pr(>|z|)
## strainRGSS 2.2874 9.8495 0.6086 3.758 0.000171 ***
## strainRGSS_35 1.7261 5.6185 0.6292 2.743 0.006080 **
## ---
## Signif. codes: 0 '***' 0.001 '**' 0.01 '*' 0.05 '.' 0.1 ' ' 1
##
## exp(coef) exp(-coef) lower .95 upper .95
## strainRGSS 9.850 0.1015 2.988 32.47
## strainRGSS_35 5.619 0.1780 1.637 19.28
##
## Concordance= 0.685 (se = 0.031 )
## Likelihood ratio test= 24.36 on 2 df, p=5e-06
## Wald test = 15.28 on 2 df, p=5e-04
## Score (logrank) test = 20.99 on 2 df, p=3e-05

# Generalized Linear Model - Females

## Call:
## survdiff(formula = Surv(time, status) ~ strain, data = female_survival_curve)
##
## N Observed Expected (O-E)^2/E (O-E)^2/V
## strain=WILD 150 16 13.6 0.4330 0.6582
## strain=RGSS 150 13 13.5 0.0222 0.0338
## strain=RGSS_35 150 12 13.9 0.2536 0.3897
##
## Chisq= 0.7 on 2 degrees of freedom, p= 0.7

## Call:
## coxph(formula = Surv(time, status) ~ strain, data = female_survival_curve)
##
## n= 450, number of events= 41
##
## coef exp(coef) se(coef) z Pr(>|z|)
## strainRGSS -0.2068 0.8132 0.3734 -0.554 0.580
## strainRGSS_35 -0.3088 0.7343 0.3819 -0.809 0.419
##
## exp(coef) exp(-coef) lower .95 upper .95
## strainRGSS 0.8132 1.230 0.3912 1.691
## strainRGSS_35 0.7343 1.362 0.3474 1.552
##
## Concordance= 0.536 (se = 0.042 )
## Likelihood ratio test= 0.7 on 2 df, p=0.7
## Wald test = 0.7 on 2 df, p=0.7
## Score (logrank) test = 0.71 on 2 df, p=0.7


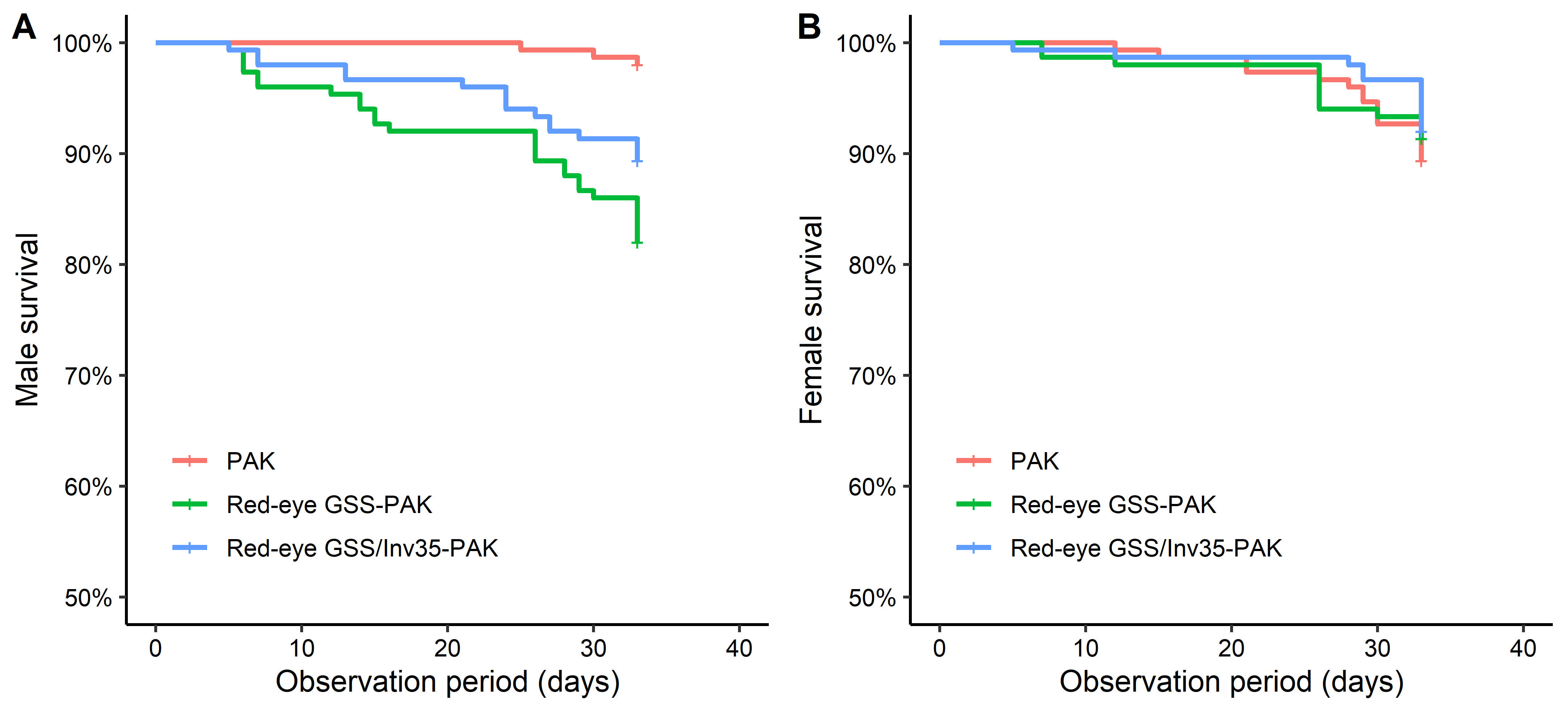


**Figure 8.** Survival rate of males (A) and females (B) of the PAK, Red-eye GSS-PAK, and Red-eye GSS/Inv35-PAK strains during the first 33 days post-emergence observation period.

# Flight Ability Test

**Table 07.** Flight ability of males of the PAK, Red-eye GSS-PAK, and Red-eye GSS/Inv35-PAK strains

| **Strain** | **Percentage of flyers** | **Standard error** |
| --- | --- | --- |
| PAK | 0.65 | 0.03 |
| Red-eye GSS-PAK | 0.73 | 0.02 |
| Red-eye GSS/Inv35-PAK | 0.82 | 0.02 |

# Generalized Linear Model - Flight Ability

##
## Call: glm(formula = flyers/total_flyers ~ strain, family = binomial(link = "logit"),
## data = flight_table,
## weights = total_flyers)
##
## Coefficients:
## (Intercept) strainRGSS strainRGSS_35
## 0.6212 0.3513 0.8981
##
## Degrees of Freedom: 8 Total (i.e. Null); 6 Residual
## Null Deviance: 30.2
## Residual Deviance: 6.249 AIC: 55.74

## Df Sum Sq Mean Sq F value Pr(>F)
## strain 2 4.591 2.2954 11.74 0.00844 **
## Residuals 6 1.173 0.1956
## ---
## Signif. codes: 0 '***' 0.001 '**' 0.01 '*' 0.05 '.' 0.1 ' ' 1

##
## Simultaneous Tests for General Linear Hypotheses
##
## Multiple Comparisons of Means: Tukey Contrasts
##
##
## Fit: glm(formula = flyers/total_flyers ~ strain, family = binomial(link = "logit"),
## data = flight_table,
## weights = total_flyers)
##
## Linear Hypotheses:
## Estimate Std. Error z value Pr(>|z|)
## RGSS - WILD == 0 0.3513 0.1719 2.044 0.1018
## RGSS_35 - WILD == 0 0.8981 0.1877 4.784 <0.001 ***
## RGSS_35 - RGSS == 0 0.5468 0.1906 2.869 0.0115 *
## ---
## Signif. codes: 0 '***' 0.001 '**' 0.01 '*' 0.05 '.' 0.1 ' ' 1
## (Adjusted p values reported -- single-step method)


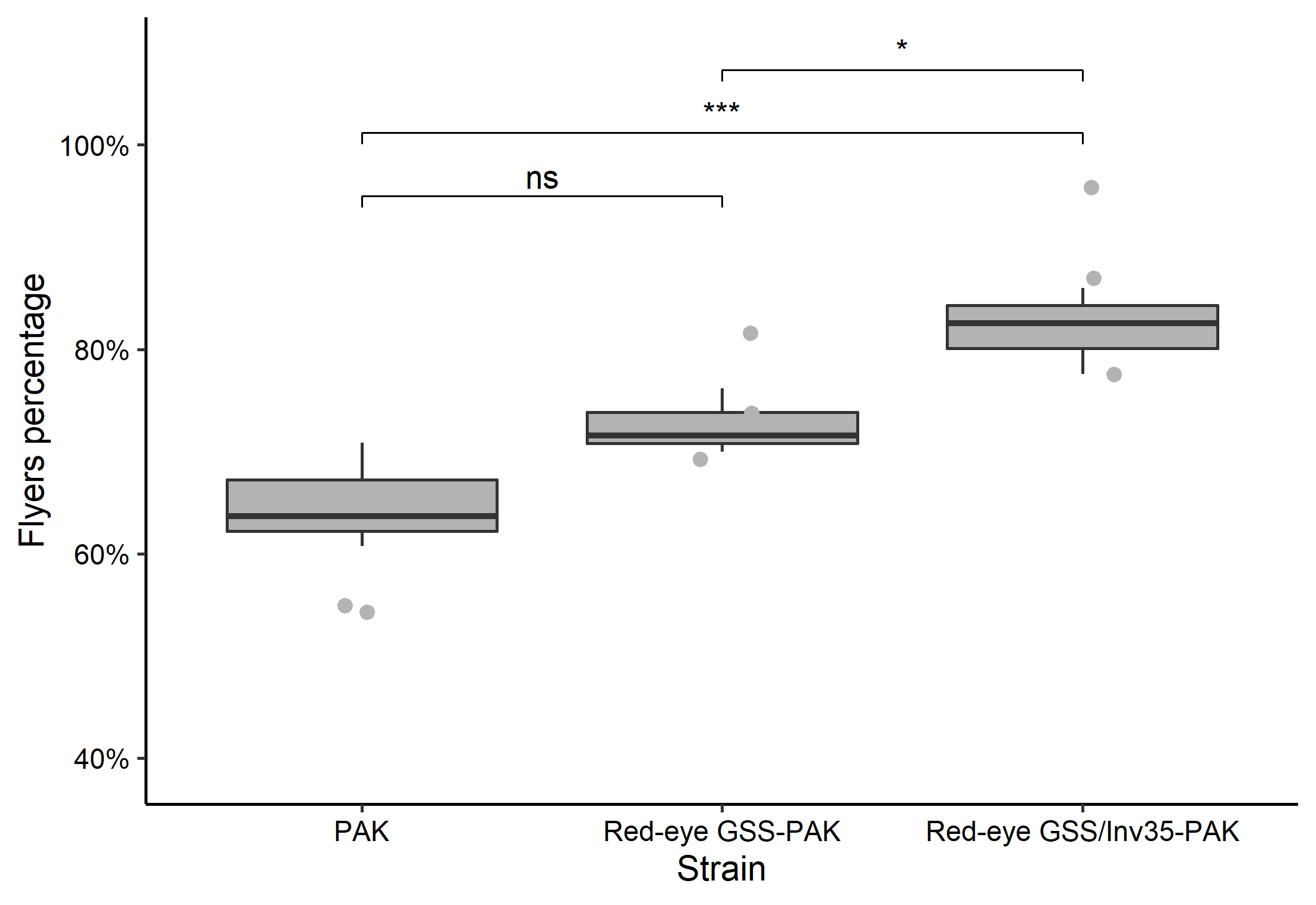


**Figure 9.** Percentage of flyers from the PAK, Red-eye GSS-PAK and Red-eye GSS/Inv35-PAK strains in the flight test

print(sessionInfo())

## R version 4.1.1 (2021-08-10)
## Platform: x86_64-w64-mingw32/x64 (64-bit)
## Running under: Windows 10 x64 (build 18363)
##
## Matrix products: default
##
## locale:
## [1] LC_COLLATE=English_United Kingdom.1252
## [2] LC_CTYPE=English_United Kingdom.1252
## [3] LC_MONETARY=English_United Kingdom.1252
## [4] LC_NUMERIC=C
## [5] LC_TIME=English_United Kingdom.1252
##
## attached base packages:
## [1] stats graphics grDevices utils datasets methods base
##
## other attached packages:
## [1] rcompanion_2.4.6 ggplotify_0.1.0 knitr_1.36
## [4] DescTools_0.99.44 multcomp_1.4-17 TH.data_1.1-0
## [7] survival_3.2-11 mvtnorm_1.1-3 ggstatsplot_0.9.0
## [10] survminer_0.4.9 MASS_7.3-54 ResourceSelection_0.3-5
## [13] ggfortify_0.4.13 scales_1.1.1 ggpubr_0.4.0
## [16] cowplot_1.1.1 ggsignif_0.6.3 forcats_0.5.1
## [19] stringr_1.4.0 dplyr_1.0.7 purrr_0.3.4
## [22] readr_2.1.1 tidyr_1.1.4 tibble_3.1.4
## [25] ggplot2_3.3.5 tidyverse_1.3.1
##
## loaded via a namespace (and not attached):
## [1] readxl_1.3.1 backports_1.3.0 plyr_1.8.6
## [4] splines_4.1.1 gmp_0.6-2.1 kSamples_1.2-9
## [7] digest_0.6.29 SuppDists_1.1-9.5 yulab.utils_0.0.4
## [10] htmltools_0.5.2 fansi_0.5.0 magrittr_2.0.1
## [13] memoise_2.0.1 paletteer_1.4.0 tzdb_0.2.0
## [16] modelr_0.1.8 matrixStats_0.61.0 sandwich_3.0-1
## [19] colorspace_2.0-2 rvest_1.0.2 haven_2.4.3
## [22] xfun_0.28 crayon_1.4.2 jsonlite_1.7.2
## [25] libcoin_1.0-9 Exact_3.1 zeallot_0.1.0
## [28] zoo_1.8-9 glue_1.4.2 gtable_0.3.0
## [31] emmeans_1.7.2 statsExpressions_1.2.0 car_3.0-12
## [34] Rmpfr_0.8-7 abind_1.4-5 DBI_1.1.2
## [37] rstatix_0.7.0 PMCMRplus_1.9.3 Rcpp_1.0.7
## [40] xtable_1.8-4 performance_0.8.0 gridGraphics_0.5-1
## [43] proxy_0.4-26 km.ci_0.5-2 stats4_4.1.1
## [46] datawizard_0.2.2 httr_1.4.2 ellipsis_0.3.2
## [49] modeltools_0.2-23 farver_2.1.0 pkgconfig_2.0.3
## [52] reshape_0.8.8 multcompView_0.1-8 dbplyr_2.1.1
## [55] utf8_1.2.2 labeling_0.4.2 tidyselect_1.1.1
## [58] rlang_0.4.11 munsell_0.5.0 cellranger_1.1.0
## [61] tools_4.1.1 cachem_1.0.6 cli_3.0.1
## [64] generics_0.1.1 broom_0.7.11 evaluate_0.14
## [67] fastmap_1.1.0 BWStest_0.2.2 yaml_2.2.1
## [70] rematch2_2.1.2 fs_1.5.2 survMisc_0.5.5
## [73] coin_1.4-2 rootSolve_1.8.2.3 nlme_3.1-152
## [76] WRS2_1.1-3 xml2_1.3.3 correlation_0.7.1
## [79] compiler_4.1.1 rstudioapi_0.13 e1071_1.7-9
## [82] reprex_2.0.1 stringi_1.7.6 highr_0.9
## [85] parameters_0.16.0 lattice_0.20-44 Matrix_1.3-4
## [88] KMsurv_0.1-5 vctrs_0.3.8 pillar_1.6.4
## [91] lifecycle_1.0.1 mc2d_0.1-21 lmtest_0.9-39
## [94] estimability_1.3 data.table_1.14.2 insight_0.15.0
## [97] lmom_2.8 patchwork_1.1.1 R6_2.5.1
## [100] gridExtra_2.3 gld_2.6.3 codetools_0.2-18
## [103] boot_1.3-28 assertthat_0.2.1 nortest_1.0-4
## [106] withr_2.4.3 mgcv_1.8-36 bayestestR_0.11.5
## [109] expm_0.999-6 parallel_4.1.1 hms_1.1.1
## [112] grid_4.1.1 coda_0.19-4 class_7.3-19
## [115] rmarkdown_2.11 carData_3.0-5 lubridate_1.8.0
